# Synthesis and in Silico Investigation of Organoselenium-Clubbed Schiff Bases as Potential M^pro^ Inhibitors for the SARS-CoV-2 Replication

**DOI:** 10.3390/life13040912

**Published:** 2023-03-30

**Authors:** Saad Shaaban, Aly Abdou, Abdulrahman G. Alhamzani, Mortaga M. Abou-Krisha, Mahmoud A. Al-Qudah, Mohamed Alaasar, Ibrahim Youssef, Tarek A. Yousef

**Affiliations:** 1 Department of Chemistry, College of Science, King Faisal University, Al-Ahsa 31982, Saudi Arabia; 2 Department of Chemistry, Faculty of Science, Mansoura University, Mansoura 35516, Egypt; 3 Department of Chemistry, Faculty of Science, Sohag University, Sohag 82524, Egypt; 4 College of Science, Chemistry Department, Imam Mohammad Ibn Saud Islamic University, Riyadh 11623, Saudi Arabia; 5 Department of Chemistry, South Valley University, Qena 83523, Egypt; 6 Department of Chemistry, Faculty of Science, Yarmouk University, Irbid 21163, Jordan; 7 Institute of Chemistry, Martin Luther University Halle-Wittenberg, 06108 Halle (Saale), Germany; 8 Department of Chemistry, Faculty of Science, Cairo University, Giza 12613, Egypt; 9 Department of Toxic and Narcotic Drug, Forensic Medicine, Mansoura Laboratory, Medicolegal Organization, Ministry of Justice, Cairo 11435, Egypt

**Keywords:** COVID-19, organoselenium, Schiff base, DFT, SARS-CoV-2, docking, ADMET

## Abstract

**Simple Summary:**

The coronavirus was declared a worldwide pandemic for the first time in December 2019. Although vaccination reduces the risk of severe illness and death, no vaccine is 100% foolproof. Recently, the COVID-19 primary protease has become a promising therapeutic target. During the preceding three years, many low molecular weight chemical libraries were tested for their potent antiviral potency against SARS-CoV-2. Many studies focused on organoselenium compounds due to their potential antiviral activities. Herein, new organoselenium-based Schiff bases were successfully synthesized and evaluated for their potential to inhibit the SARS-CoV-2 M^pro^ main protease, which is essential for virus replication.

**Abstract:**

Since the first report of the organoselenium compound, ebselen, as a potent inhibitor of the SARS-CoV-2 M^pro^ main protease by Z. Jin et al. (Nature, 2020), different OSe analogs have been developed and evaluated for their anti-COVID-19 activities. Herein, organoselenium-clubbed Schiff bases were synthesized in good yields (up to 87%) and characterized using different spectroscopic techniques. Their geometries were studied by DFT using the B3LYP/6–311 (d, p) approach. Ten FDA-approved drugs targeting COVID-19 were used as model pharmacophores to interpret the binding requirements of COVID-19 inhibitors. The antiviral efficiency of the novel organoselenium compounds was assessed by molecular docking against the 6LU7 protein to investigate their possible interactions. Our results showed that the COVID-19 primary protease bound to organoselenium ligands with high binding energy scores ranging from −8.19 to −7.33 Kcal/mol for **4c** and **4a** to −6.10 to −6.20 Kcal/mol for **6b** and **6a**. Furthermore, the docking data showed that **4c** and **4a** are good M^pro^ inhibitors. Moreover, the drug-likeness studies, including Lipinski’s rule and ADMET properties, were also assessed. Interestingly, the organoselenium candidates manifested solid pharmacokinetic qualities in the ADMET studies. Overall, the results demonstrated that the organoselenium-based Schiff bases might serve as possible drugs for the COVID-19 epidemic.

## 1. Introduction

Organoselenium (OSe) agents have gained considerable concern due to their diverse applications in pharmaceutical chemistry [1,2]. The selenium (Se) element is present in almost all organisms as a part of different selenoenzymes (e.g., glutathione peroxidase (GPX) and thioredoxin reductases (TrxR)) [1,3]. Moreover, Se is crucial for protecting cells against oxidative damage [4]. It is also essential for the regular function of the immune system via tolerating resistance against viral infection. On the other hand, Se deficiency is engaged with viral infection progression and disease severity [5]. Within this context, it also boosts the host’s immunity via activating GPX and TrxR and stimulating the intracellular redox status [6].

**Scheme 1:**
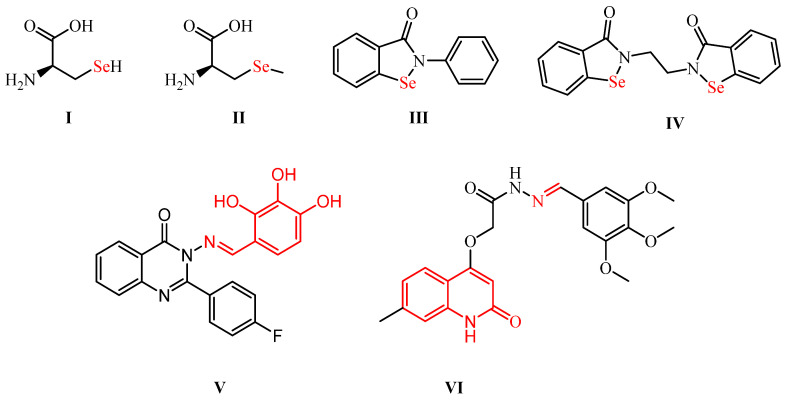
Structures of potential OSe compounds (**I**–**IV**) and Schiff bases (**V**,**VI**) for SARS-CoV-2 therapy.

Furthermore, Se has a bigger size, lower electronegativity, and higher polarizability than its analogs: sulfur, nitrogen, and phosphorous atoms [1,7]. Therefore, OSe compounds are considered stronger nucleophiles, which is also why OSe enhances catalytic activity [1]. Additionally, different OSe agents inhibit oxidative stress-based diseases by free radical (e.g., oxygen and nitrogen (scavenging [1,5]. Se is the main constituent of various naturally occurring and biologically active compounds, such as selenoproteins as part of selenocysteine (**I**) and selenomethionine (**II**) amino acids [1,8]. Moreover, the selenocyanates BSeC (**III**) and pXSeC (**IV**) exhibit potential chemoprotective activities against colon and lung cancers (Scheme 1) [9]. Additionally, ethaselen (**V**) shows interesting TrxR inhibitory activities and is latterly being evaluated in clinical trial II [5,7,10].

Furthermore, ebselen (**VI**) is one of the most researched OSe agents with exciting GPX-like properties and has currently reached clinical phase II for hypo/manic treatment (Scheme 1) [1,11]. Additionally, Jin et al. reported ebselen over more than ten thousand compounds as a SARS-CoV-2 M^pro^ lead inhibitor [1,12]. The promising antiviral activity of ebselen opened the door for the potential investigation of OSe compounds as possible M^pro^ inhibitors [12].

The primary protease of SARS-CoV-2 is an essential component in viral replication. Research towards a treatment for COVID-19 centers on this protein. The binding affinity and structure of protein–drug complexes also play critical roles in elucidating the molecular process underlying drug development. The significance of developing alternate, more scalable therapies remains high since COVID-19 is not an uncommon condition. Most notably, a novel candidate that blocks the interaction between the COVID-19 major protease and the cell surface angiotensin converting enzyme-2 are highly desired. These considerations prompted us to perform an in-silico search, for the interaction above, between the primary protease active site and the complex named in the subheading. Lastly, the use of computer simulations to predict antibiotic efficacy was investigated.

On the other hand, Schiff bases provide several benefits that have led to their extensive usage in the chemical, biological, and medicinal fields. These compounds have various biological activities, including antioxidant, antifungal, and anticancer properties [13]. Within this context, tri-hydroxyphenyl Schiff bases have shown potent activity against the SARS-CoV-2 virus [14]. Furthermore, quinazoline Schiff bases showed antiviral activity against different virus strains, for instance, influenza, herpes, and feline coronaviruses.

Herein, we aim to develop novel OSe-clubbed Schiff bases and evaluate their possible antiviral activities to inhibit the M^pro^ essential for SARS-CoV-2 replication using density function theory, electrostatic potential, and molecular frontier orbital studies. In addition, the drug-likeness was investigated by employing molecular docking and ADMET properties.

## 2. Materials and Methods

Melting points were recorded in degrees centigrade using a Gallen-Kamp instrument. The IR spectra were recorded (KBr, ύ cm^−1^) at King Faisal University on a Mattson 5000 FTIR spectrophotometer. The ^1^H-NMR and the ^13^C-NMR spectra were measured using a Varian spectrophotometer at 500 MHz, employing the TMS internal reference and as the solvents. The chemical shifts (*δ*), in parts per million, were recorded with respect to the residual peak of solvents. Compounds **2**, **3**, and **5** were synthesized according to our literature reports (see detailed experimental procedures in the Supporting Materials) [15].

### 2.1. Synthesis and Characterization

General procedure for the synthesis of OSe mealanilic **4a**–**c** derivatives

To a solution of diselenide amine **3** (1 mmol) in ethanol (15 mL), aromatic aldehyde (2.2 mmol) was added, and the mixture was refluxed for 6 hrs. After cooling, the formed crystals were filtered and washed with cold ethanol. The obtained Schiff bases were recrystallized from ethanol.

General procedure for the synthesis of OSe mealanilic **6a**–**c** derivatives

To a solution of methylselenoamine **5** (1 mmol) in ethanol (15 mL), aromatic aldehyde (1.2 mmol) was added, and the mixture was refluxed for 6 hrs. After cooling, the formed crystals were filtered and washed with cold ethanol. The obtained Schiff bases were recrystallized from ethanol.

*N,N′-(diselanediylbis(4,1-phenylene))bis(1-(4-fluorophenyl)methanimine) (**4a**)*

Compound **4a** was synthesized from diselenide **3** (1 mmol, 342 mg) and 4-fluoro benzaldehyde (1.2 mmol, 148.8 mg). The reaction was followed by TLC (EtOAc/hexane1:3; Rf = 0.33) and isolated as a yellow solid with an 80% yield (445 mg), and its MP was 139–140 °C. IR (FT-IR, cm^−1^): 2930, 1619, 1150, 1018, 997; ^1^H NMR (500 MHz, DMSO-d_6_) δ 8.63 (s, 2H, HC=N), 7.98 (dd, *J* = 8.7, 5.7 Hz, 4H, Ar-H), 7.66 (t, *J* = 9.7 Hz, 4H, Ar-H), 7.35 (t, *J* = 8.8 Hz, 4H, Ar-H), 7.24 (d, *J* = 8.5 Hz, 4H, Ar-H); ^13^C NMR (126 MHz, DMSO-d_6_) δ 165.54, 163.55, 160.54, 151.66, 133.00, 131.61, 131.54, 127.72, 122.59, 116.33; MS (ESI): *m/z* = found 555.2 [M^+^]; calcd. 555.8 [M^+^]. Anal. calcd. for C_26_H_18_F_2_N_2_Se_2_ (554.98) C, 56.33; H, 3.27; N, 5.05. Found C, 56.36; H, 3.30; N, 5.09.

*N,N′-(diselanediylbis(4,1-phenylene))bis(1-(4-bromophenyl)methanimine) (**4b**)*

Compound **4b** was synthesized from diselenide **3** (1 mmol, 342 mg) and 4-bromobenzaldehyde (1.2 mmol, 222 mg). The reaction was followed by TLC (EtOAc/hexane 1:3; Rf = 0.34) and isolated as a yellow solid with an 87% yield (587.25 mg), and its MP was 175–176 °C. IR (FT-IR, cm^−1^): 3025, 1618, 1340, 1151, 1021, 997; ^1^H NMR (500 MHz, DMSO-d_6_) δ 8.63 (s, 2H, HC=N), 7.86 (d, *J* = 8.5 Hz, 4H, Ar-H), 7.73 (d, *J* = 8.5 Hz, 4H, Ar-H), 7.68 (d, *J* = 8.5 Hz, 4H, Ar-H), 7.26 (d, *J* = 8.5 Hz, 4H, Ar-H); ^13^C NMR (126 MHz, DMSO-d_6_) δ 160.75, 151.43, 135.49, 132.98, 132.38, 131.01, 127.99, 125.69, 122.64; MS (ESI): *m/z* = found 677.1 [M^+^ + 1]; calcd. 675.8 [M^+^]. Anal. calcd. for C_26_H_18_Br_2_N_2_Se_2_ (676.19) C, 46.18; H, 2.68; N, 4.14. Found C, 46.15; H, 2.64; N, 4.19.

*N,N′-(diselanediylbis(4,1-phenylene))bis(1-(2-nitrophenyl)methanimine) (**4c**)*

Compound **4c** was synthesized from diselenide **3** (1 mmol, 342 mg) and 2-nitrobenzaldehyde (1.2 mmol, 182 mg). The reaction was followed by TLC (EtOAc/hexane 1:3; Rf = 0.30) and isolated as a yellow solid with an 85% yield (573.5 mg), and its MP was 136–137 °C. IR (FT-IR, cm^−1^): 3099, 1612, 1517, 1335, 1022, 1006; ^1^H NMR (500 MHz, DMSO-d_6_) δ 8.90–8.85 (s, 2H, HC=N), 8.16 (dd, *J* = 7.8, 1.3 Hz, 2H, Ar-H), 8.11 (dd, *J* = 8.1, 1.0 Hz, 2H, Ar-H), 7.86 (t, *J* = 7.6 Hz, 2H, Ar-H), 7.77 (t, *J* = 8.1, 1.4 Hz, 2H, Ar-H), 7.74–7.70 (m, 4H, Ar-H), 7.31–7.23 (m, 4H, Ar-H); ^13^C NMR (126 MHz, DMSO-d_6_) δ 157.91, 151.01, 149.72, 134.21, 132.99, 132.48, 130.34, 130.03, 128.71, 124.98, 122.71; MS (ESI): *m/z* = found 609.2 [M^+^], 633.2 [M^+^ + Na]; calcd. 609.9 [M^+^]. Anal. calcd. for C_26_H_18_N_4_O_4_Se_2_ (609.98) C, 51.33; H, 2.98; N, 9.21. Found C, 51.34; H, 2.97; N, 9.23.

*1-(4-fluorophenyl)-N-(4-(methylselanyl)phenyl)methanimine (**6a**)*

Compound **6a** was synthesized from methylselenoamine **5** (1 mmol, 187 mg) and 4-fluoro benzaldehyde (1.2 mmol, 148.8 mg). The reaction was followed by TLC (EtOAc/hexane 1:3; Rf = 0.35) and isolated as a yellow solid with an 86% yield (252 mg), and its MP was 138–139 °C. IR (FT-IR, cm^−1^): 3000, 1616, 1418, 1150, 1020, 997; ^1^H NMR (500 MHz, DMSO-d_6_) δ 8.64 (s, 1H, HC=N), 7.91–7.84 (m, 2H, Ar-H), 7.75–7.68 (m, 2H, Ar-H), 7.50–7.41 (m, 2H, Ar-H), 7.27–7.19 (m, 2H, Ar-H), 2.39 (s, 3H, CH_3_); ^13^C NMR (126 MHz, DMSO-d_6_) δ 159.56, 149.37, 135.68, 132.34, 130.88, 130.81, 129.91, 125.41, 122.45, 7.21; MS (ESI): *m/z* = found 310.2 [M^+^ + NH_4_]; calcd. 293.0 [M^+^]. Anal. calcd. for C_14_H_12_FNSe (293.01) C, 57.54; H, 4.14; N, 4.79. Found C, 57.49; H, 4.12; N, 4.72.

*1-(((4-(methylselanyl)phenyl)imino)methyl)naphthalen-2-ol (**6b**)*

Compound **6b** was synthesized from methylselenoamine **5** (1 mmol, 187 mg) and 2-hydroxy-1-naphthaldehyde (1.2 mmol, 206 mg). The reaction was followed by TLC (EtOAc/hexane 1:3; Rf = 0.31) and isolated as a yellow solid with a 54% yield (184 mg), and its MP was 99–100 °C. IR (FT-IR, cm^−1^): 3109, 2961, 1617, 1471, 1119, 1019, 996; ^1^H NMR (500 MHz, DMSO-d_6_) δ 9.65 (s, 1H, HC=N), 8.48 (d, *J* = 8.4 Hz, 1H, Ar-H), 7.91 (d, *J* = 9.1 Hz, 1H, Ar-H), 7.78 (d, *J* = 7.8 Hz, 1H, Ar-H), 7.60–7.47 (m, 5H, Ar-H), 7.34 (t, *J* = 7.4 Hz, 1H, Ar-H), 7.00 (d, *J* = 9.1 Hz, 1H, Ar-H), 2.39 (s, 3H, CH_3_); ^13^C NMR (126 MHz, DMSO-d_6_) δ 170.57, 155.65, 142.47, 137.18, 133.53, 131.02, 130.33, 129.44, 128.51, 127.14, 123.92, 122.48, 121.76, 120.83, 109.04, 7.23; MS (ESI): *m/z* = found 340.1 [M^+^ − H], 342.2 [M^+^ + H]; calcd. 341.0 [M^+^]. Anal. calcd. for C_18_H_15_NOSe (341.03) C, 63.53; H, 4.44; N, 4.12. Found C, 63.58; H, 4.42; N, 4.17.

*N-(4-(methylselanyl)phenyl)-1-(2-nitrophenyl)methanimine (**6c**)*

Compound **6c** was synthesized from methylselenoamine **5** (1 mmol, 187 mg) and 2-nitrobenzaldehyde (1.2 mmol, 182 mg). The reaction was followed by TLC (EtOAc/hexane 1:3; Rf = 0.30) and isolated as a yellow solid with a 57% yield (182 mg), and its MP was 161–162 °C. IR (FT-IR, cm^−1^): 2923, 1658, 1342, 1151, 1019, 1008; ^1^H NMR (500 MHz, DMSO-d_6_) δ 8.88 (s, 1H, HC=N), 8.16 (d, *J* = 6.7, 3.3 Hz, 1H, Ar-H), 8.10 (d, *J* = 8.1 Hz, 1H, Ar-H), 7.85 (t, *J* = 7.6 Hz, 1H, Ar-H), 7.78–7.73 (m, 1H, Ar-H), 7.47 (d, *J* = 8.4 Hz, 2H, Ar-H), 7.23 (d, *J* = 8.5 Hz, 2H, Ar-H), 2.40–2.34 (s, 3H, CH_3_); ^13^C NMR (126 MHz, DMSO-d_6_) δ 156.67, 149.66, 149.00, 134.16, 132.26, 130.79, 130.52, 129.92, 124.96, 122.52, 7.17; MS (ESI): *m/z* = found 321.2 [M^+^ + H]; calcd. 320.0 [M^+^]. Anal. calcd. for C_14_H_12_N_2_O_2_Se (320.01) C, 52.67; H, 3.79; N, 8.78. Found C, 52.70; H, 3.74; N, 8.76.

### 2.2. Computational Calculations

The details of the used protocol for the computational calculations and DFT calculations using Gaussian 09 [16], in addition to pharmacophore analysis, molecular docking investigation using Molecular Operating Software (MOE) [17], and drug-likeness properties, are listed in the Supplementary Information.

## 3. Results

### 3.1. Design and Synthesis of the Organoselenium Compounds

**Scheme 2:**
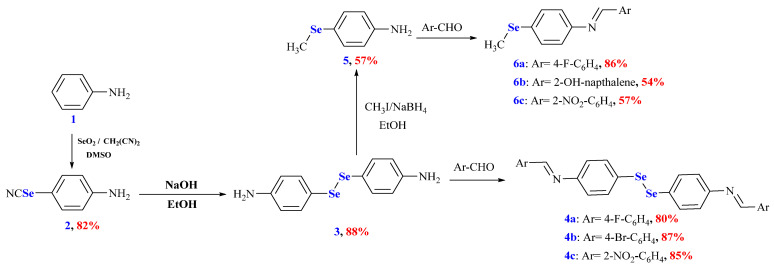
Synthesis of OSe compounds **2**–**6.** Reagents and conditions: (i) selenocyanate amine **2** was obtained in an 82% yield from PhNH_2_ (24 mmol), SeO_2_ (3.2 mmol), CH_2_(CN)_2_ (1.6 mmol), and DMSO (5 mL); (ii) diselenide **3** was obtained in an 88% yield from selenocyanate amine **2** (4 mmol), NaOH (6 mmol), and MeOH (20 mL); (iii) diselenide bis Schiff bases **4a**–**c** were obtained from the reaction of diselenide **3** (1 mmol) and appropriate aldehyde (2.2 mmol); (iv) Ose amine **5** was obtained in a 57% yield from diselenide **3** (2 mmol), NaOH (2 mmol), NaBH_4_ (6 mmol), CH_3_I (4.4 mmol), and EtOH (25 mL); (v) organic selenide Schiff bases **6a**–**c** were obtained from the reaction of Ose amine **5** (1 mmol) and appropriate aldehyde (1.1 mmol).

The OSe agents manifested immense activities and potential applications. Therefore, their synthesis is highly required. Unfortunately, developing OSe compounds is commonly associated with several synthetic challenges. These include using hazardous, expensive reagents such as NaSeH, Cu_2_Se, and Na_2_SeSO_3_ [1,18]. On the other hand, Schiff bases have manifested significant applications in pharmaceutical and medicinal chemistry owing to their broad spectrum of pharmacological properties (e.g., antitubercular, anti-inflammatory, and anticancer). Accordingly, herein, we aim to combine Schiff bases and OSe in one scaffold and, in silico, investigate their potential for SARS-CoV-2 M^pro^ inhibitions [8]. Selenocyanate **2** and diselenide **3** are considered versatile precursors and building blocks of Se-based architectures. Selenocyanate amine **2** was obtained from an aniline reaction with triselenium dicyanide in an 82% yield. The alkaline hydrolysis of selenocyanate **2** afforded the corresponding diselenide diamine **3** in an 88% yield. The reduction of diselenide **3** using NaBH_4_ and its subsequent reaction with CH_3_I furnished 4-(methylselanyl)aniline (**5**) in a 57% yield. The reaction of diamine **3** with different aromatic aldehydes, namely 4-fluoro benzaldehyde, 4-bromobenzaldehyde, and 2-nitrobenzaldehyde, afforded the corresponding Schiff bases **4a**–**c** in 80%, 87%, and 85% yields, respectively. Similarly, the reaction of 4-(methylselanyl)aniline (**5**) with different aromatic aldehydes, namely 4-fluoro benzaldehyde, 2-hydroxy naphthaldehyde, and 2-nitrobenzaldehyde, afforded the corresponding Schiff bases **6a**–**c** in 80%, 87%, and 85% yields, respectively (Scheme 2).

### 3.2. DFT Calculations

Density functional theory (DFT) is a powerful computational tool for quantitatively predicting and describing biomolecular processes. With the help of DFT, it is possible to predict physical properties with a high degree of accuracy. Quantum chemical parameters such as LUMO Energy (energy of the lowest unoccupied molecular orbital), HOMO Energy (energy of the highest occupied molecular orbital), the gap’s energy (ΔE = E_HOMO_ – E_LUMO_), global electrophilicity (ω), electronegativity (χ), softness (σ), and chemical hardness (η) all influence the electronic interaction of the molecule’s atoms with the target [19,20].

#### 3.2.1. Geometry Optimization

**Figure 1:**
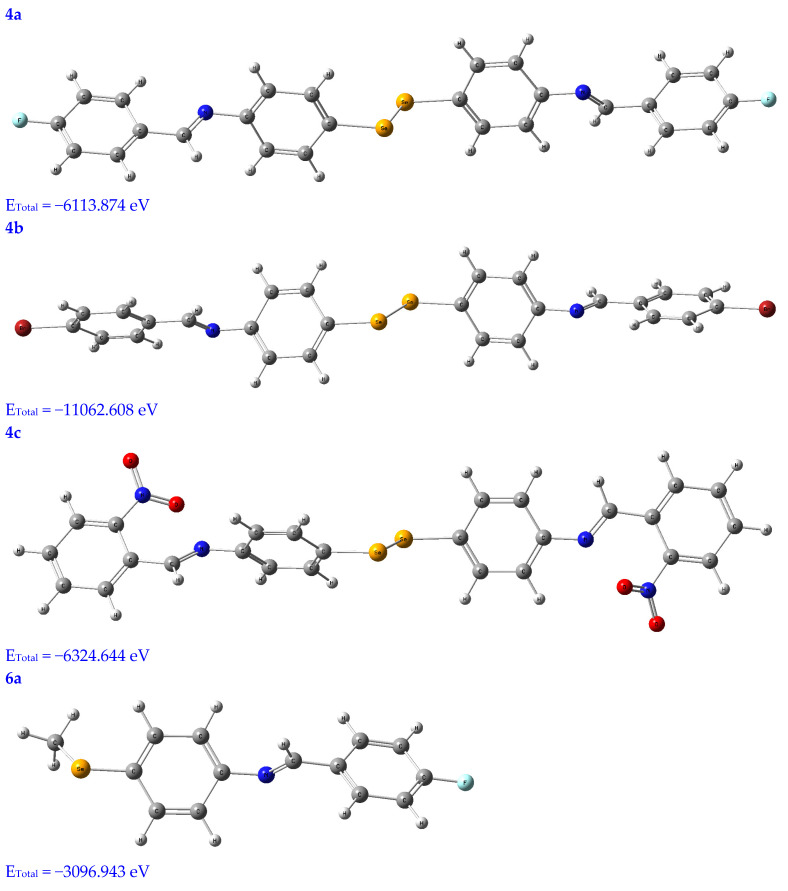

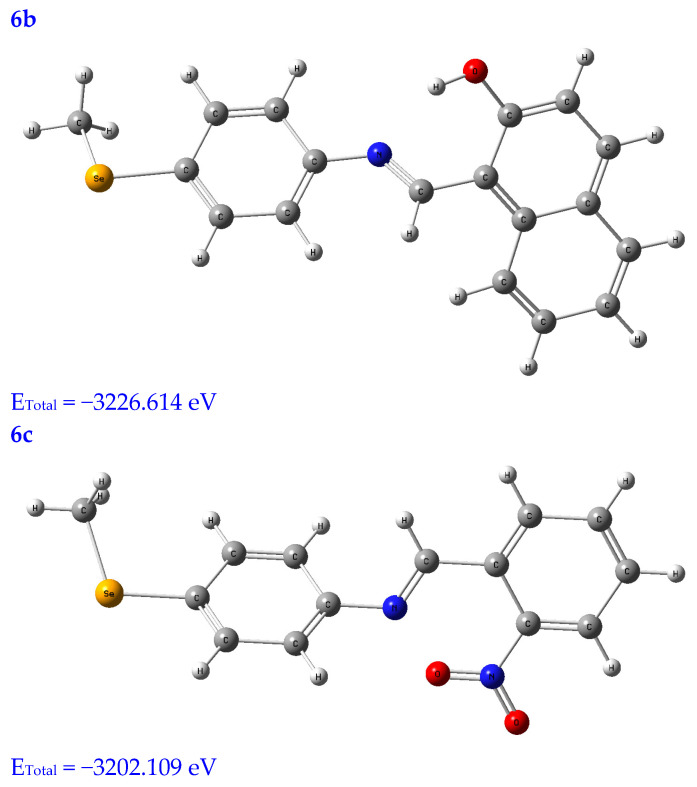
Optimized structures of the tested compounds (**4a**, **4b**, **4c**, **6a**, **6b**, and **6c**) with atom labels using DFT/B3LYP with a 6-311G (d, p) basis set.

Molecular modeling is a popular tool for structural research and provides insight into the compound’s three-dimensional shape and could be used to find the energy-minimized conformation [20]. The structural characterization of the title compounds increasingly relies on molecular modeling without X-ray crystal data. Thus, in Figure 1, the molecules under study were optimized using a B3LYB/6-311 (d, p) basis set.

#### 3.2.2. Frontier Molecular Orbital Analysis (FMO) Analysis

**Figure 2:**
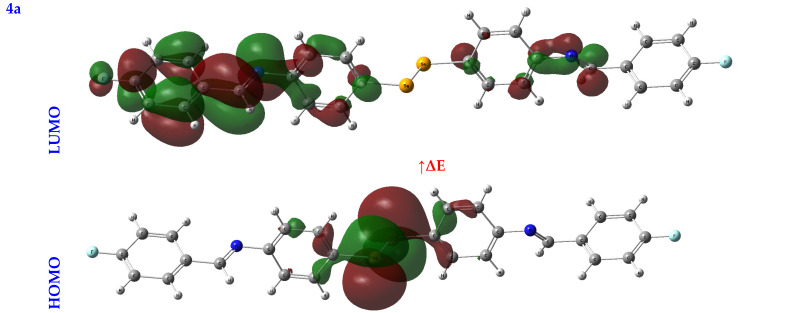

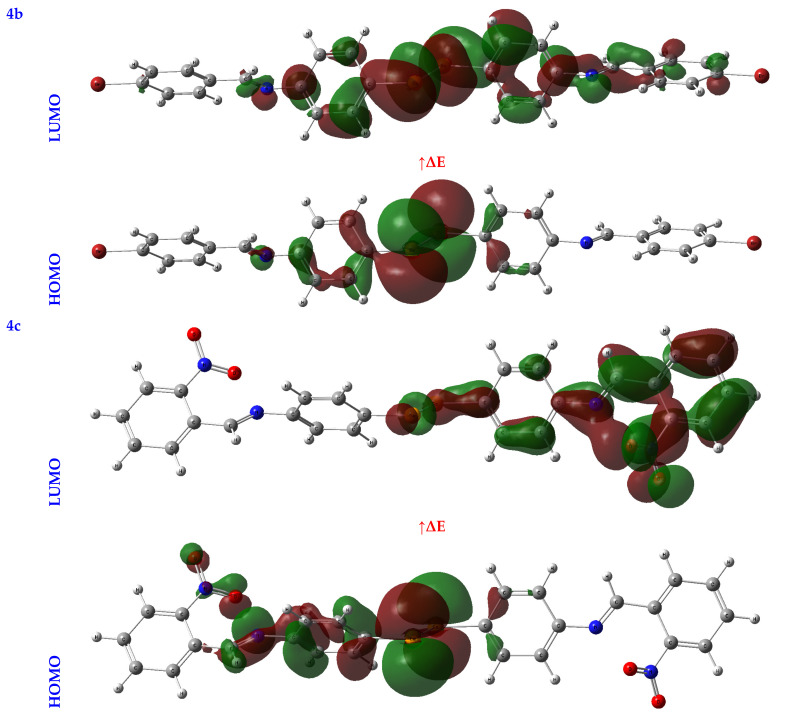

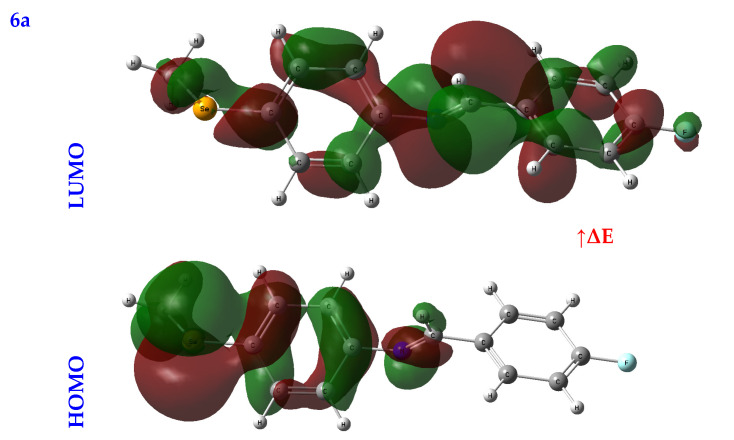

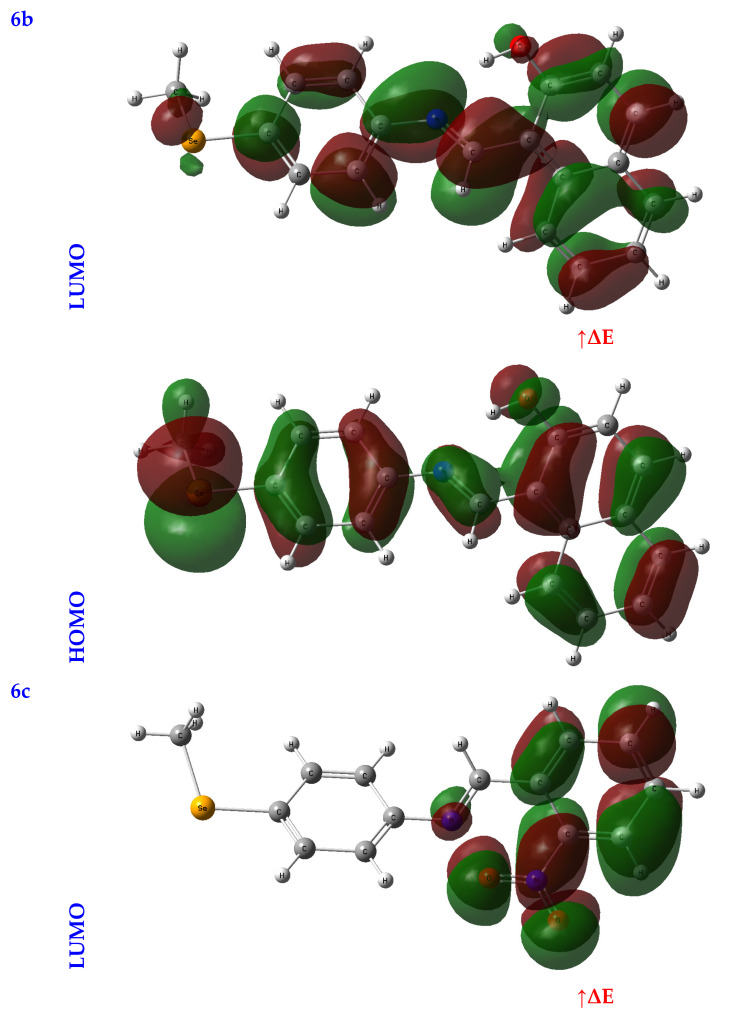

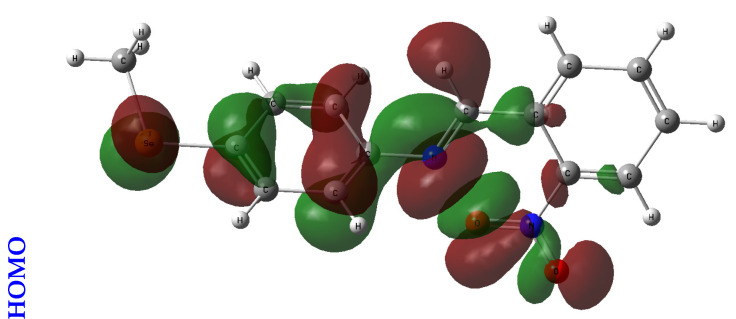
HOMO-LUMO orbitals of the tested compounds (**4a**, **4b**, **4c**, **6a**, **6b**, and **6c**) with atom labels using DFT/B3LYP with a 6-311G (d, p) basis set.

An orbital analysis is very useful for understanding all chemical processes. Molecular orbitals, also known as MOs, are essential for better understanding chemical processes and electrical and electronic properties. In the vicinity of 1952, Fukui put out the border orbital hypothesis, which establishes a connection between the properties of the HOMO and LUMO molecular orbitals and reactivity [21,22,23]. Figure 2 displays a scribbled version of the molecular orbital diagrams for the HOMO and LUMO states.

Critical activity parameters calculated from the HOMO energy (EHOMO) and the LUMO energy (E_LUMO_) may be used by the DFT calculation to forecast the biological potency of the compounds [24]. As a direct result, the molecule’s electrons are spread out relatively evenly. Therefore, energy gaps (ΔEs), ionization potentials (IPs), electron affinities (EAs), electronegativity (χ), chemical potentials (CPs), hardness (η), softness (σ), electrophilicity (ω), and nucleophilicity (Nu) may all be determined from LUMO-HOMO energies [25], Table 1.

#### 3.2.3. Global Reactive Indices

In studying a molecule’s kinetic stability, biological activity, polarizability, chemical reactivity, and hardness–softness, HOMO-LUMO energies are an appealing area to investigate as a potential source of information. The HOMO was the most distant electron orbital; its function was to donate electrons. At the same time, the LUMO described the electron acceptor for the innermost orbital that was vacant. Because of this, a molecule’s HOMO orbitals and the LUMO orbitals define its reactivity with electrophiles and nucleophiles, respectively [26]. The higher value of E_HOMO_ demonstrated that it was simpler for electrons to be transferred from the substrate to the target proteins, as illustrated by the E_HOMO_ and E_LUMO_ found in Table 1. Conversely, a smaller E_LUMO_ value suggested a simpler electron transfer between the substrate and target proteins, Table 1.

A molecule’s reactivity may be estimated by measuring its energy gap (ΔE) or the difference between its lowest unoccupied orbital (E_LUMO_) and highest occupied orbital (E_HOMO_) [27,28]. Because of this, a lower value for e indicates that the molecule is more receptive to docking. As a result, the total reactivity of the compounds that were investigated has the following order: **4c** > **4a** > **4b** > **6c** > **6a** > **6b**.

The chemical reactivity ranking is also determined by two other important factors: the hardness and softness of the substance. The tendency of a molecule to connect with another molecule may be explained by the hard-soft acid-base (HSAB) rule [29]. The rule states that weak acids are more likely to react with weak bases, whereas strong acids are more likely to react with strong bases. Cells, proteins, and other biological macromolecules fall within soft biological molecules. Because of this, soft molecules are more likely to interact with biological molecules than hard ones. Because of this, the level of physical activity rises as the level of softness increases while the level of hardness falls [30]. As a result, the following should be the sequence in which reactions take place: **4c** > **4a** > **4b** > **6c** > **6a** > **6b**, Table 1.

The chemical potential’s negative value provided evidence of the stable nature of the identified compounds. On the contrary, the electrophilic activity is enhanced by the high electrophilicity index and the low chemical potential [31].

#### 3.2.4. The Molecular Electrostatic Potential (MEP) Diagram

The rate at which a protein binds to a substrate is significantly affected by the partial charges on both the protein and the substrate. To better understand the topological and structural characteristics of substrates in three dimensions, the molecular electrostatic potential (MEP) diagram may be used. The MEP test can be used to rank the relative importance of the nuclear and electron effects on molecular geometry [32].

Every value in an MEP diagram is coded with a different color, from blue to red and everywhere in between. For example, electrophilic and nucleophilic reactivity corresponds to the MEP’s blue and red portions. Red indicates places with a negative electrical charge (i.e., those areas where accepting an electrophile is most favorable). 

**Figure 3:**
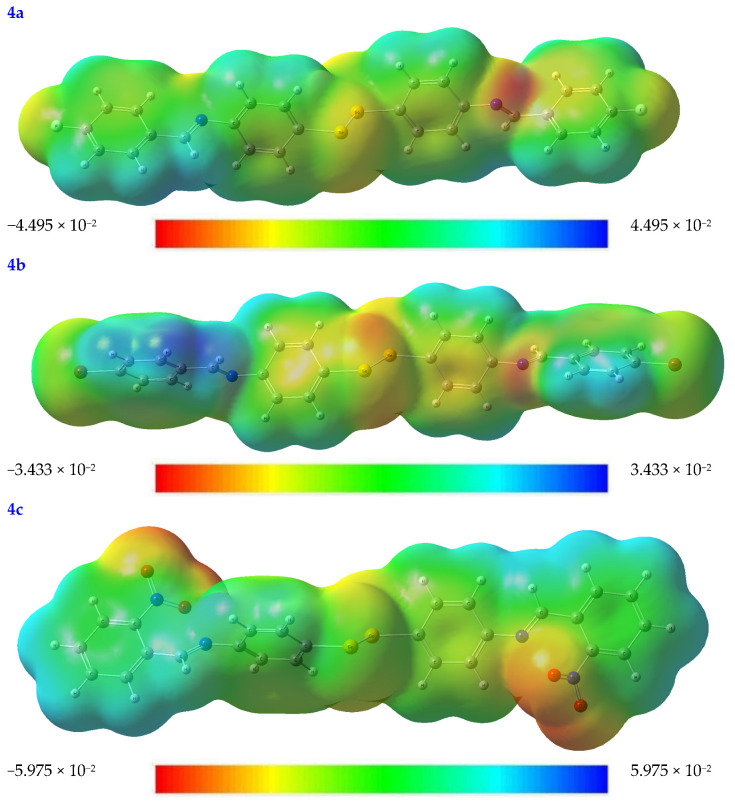

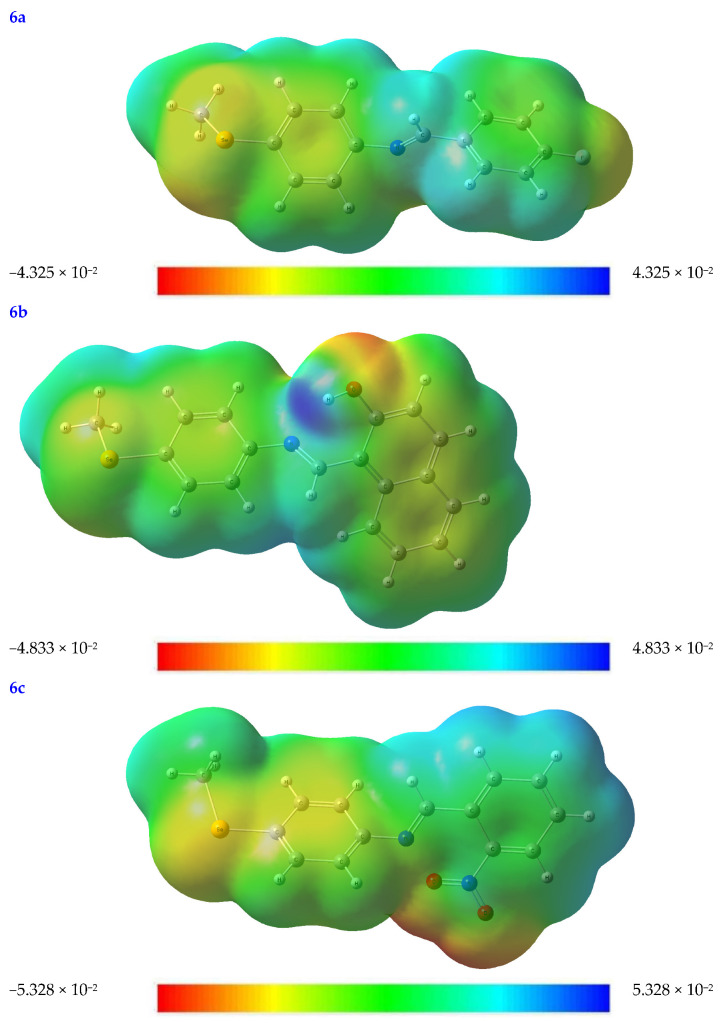
The molecular electrostatic potential (MEP) map of the optimized structures of the tested compounds (**4a**, **4b**, **4c**, **6a**, **6b**, and **6c**) with atom labels using DFT/B3LYP with a 6-311G (d, p) basis set.

At the B3LYB/6-311 (d, p) basis sets, the molecular electrostatic potential (MEP) map is mapped out for the compounds under investigations (see Figure 3). The oxygen, nitrogen, and Se moieties are where most negative regions (shown in red-orange), caused by the availability of electrons, may be found in the substrates under consideration. Because of this, these locations are also excellent candidates for attack by electrophiles. In contrast, more positive regions are shown in blue. This is because they are mainly oriented toward the hydrogen and carbon atoms, which may serve as an H-bond donor in protein–substrate intermolecular interactions (see Figure 3).

### 3.3. Drug-likeness Screening

When creating new drugs, the drug-likeness features outlined in Lipinski’s rule of five are necessary [33,34]. For a substrate to be called drug-like, it must satisfy the following criteria: it must have a molecular weight (M.wt) of 500, the number of H-bond acceptors (NHBAs) must be 10, the number of H-bond donors (NHBDs) must be five, and its lipophilicity must be expressed as log P 5 [35]. All the compounds in question adhere to the Lipinski rule of five, found in Supplementary Information, Table S1 in supplementary, which provides strong evidence that they are suitable for use as medications.

Martin [36] devised the Abbot Bioavailability Score (ABS), which assumes that at least 10% of the compounds would likely be bioavailable in rats. If the Lipinski rule of five is followed precisely, the ABS for the compounds is calculated to be 0.55; otherwise, **4a** and **4b** were estimated to be 0.17. Because the subject compounds have an ABS value of 0.55, these compounds have satisfied the prerequisite for drug similarity.

The water solubility, measured in logs, and the gastrointestinal absorption permeability, measured in G.I., are connected to the criteria for drug-likeness. They are also used to assess the early stage of oral bioavailability. The logKp value for skin permeability is within the typical range of −5.08 to −5.89. The importance of the log for the compounds in question varies from −3.77 to −8.02, which suggests that the compounds have a low solubility in water [37] and good absorption in the gut (except for 7 and 9) [38]. The ADME results showed that all the identified drugs had significant absorption levels in the gastrointestinal tract. No single item on the list can cross the blood–brain barrier (BBB) or act as a substrate for P-glycoprotein (Pgp).

In addition, the level of synthetic accessibility of the substances under investigation was evaluated. Its score ranges from 1 (very easy) to 10 (very difficult), and it is derived from 1024 different fragment contributions that are restricted by size and complexity [39]. The synthetic accessibility, which ranged from 2.69 to 3.91, indicated that the compounds in question were simple to synthesize and projected that they would be available in good yield, which was in line with the experiment’s results.

**Figure 4:**
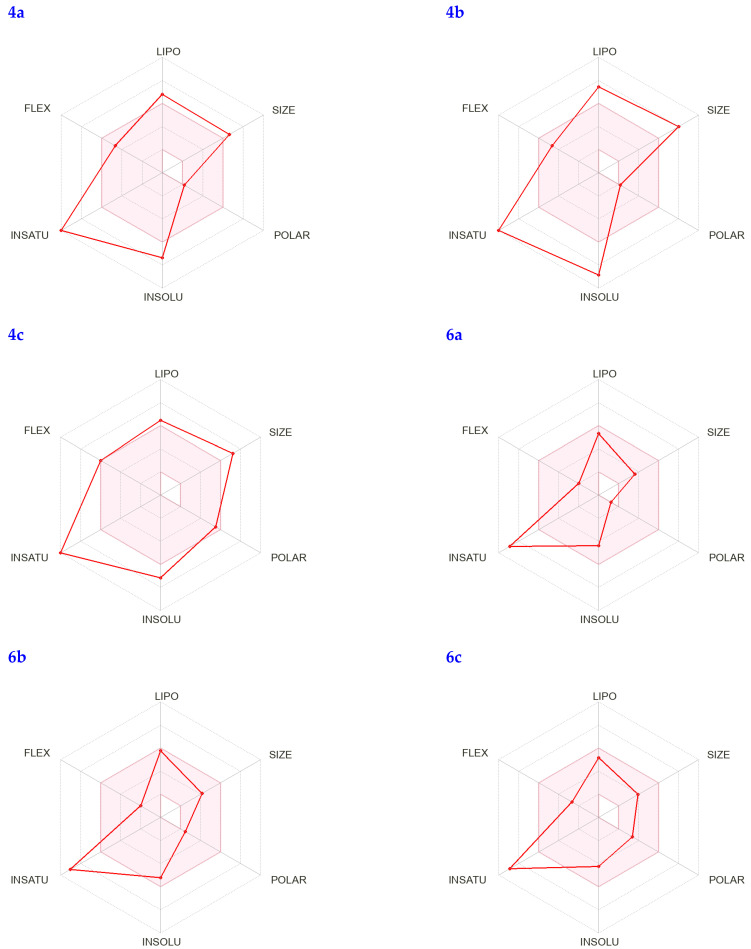
The colored zone is the suitable physicochemical space for oral bioavailability; LIPO (Lipophilicity), SIZE: (M.wt), POLAR (Polarity), INSOLU (Insolubility), INSATU (Instauration), and FLEX (Flexibility).

In addition, the bioavailability radar can quickly determine whether a chemical behaves like a medication by analyzing factors such as its saturation, lipophilicity, polarity, size, solubility, and flexibility. The visual representation of these physicochemical properties for the identified compounds can be seen in Figure 4; each characteristic’s pink zone inside the hexagon reflects the best range for that feature.

**Figure 5:**
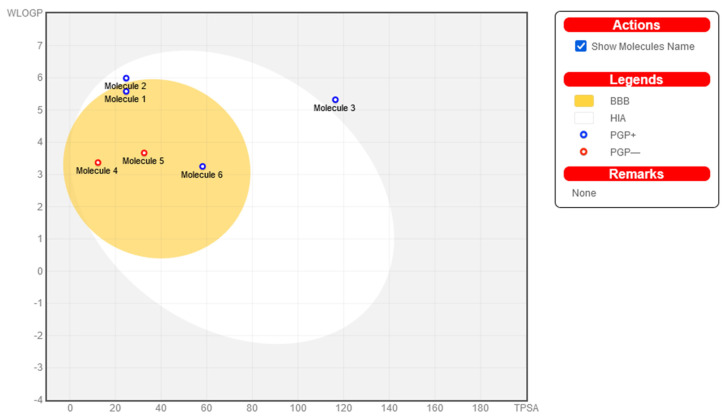
The bioavailability radars of newly synthesized derivatives; molecule 1 (**4a**), molecule 2 (**4b**), molecule 3 (**4c**), molecule 4 (**6a**), molecule 5 (**6b**), and molecule 6 (**6c**).

The Brain or Intestinal Estimated permeation prediction model, also known as BOILED-Egg, was developed by the Swiss ADME online web server. This model was used to estimate the amount of absorption of nine steroids in the brain and the gastrointestinal tract. Nine steroids are given in Figure 5. According to the data, all the substances may be taken in by the mouth and have good absorption in the gastrointestinal tract. As a result, no chemical can make it past the BBB, which shields its inhabitants from any potentially harmful effects on the CNS. Furthermore, P-glycoprotein sensitivity is not an issue since none of the substances are present. As a result, we should expect very little to no opposition.

### 3.4. Pharmacokinetic Properties Analysis

The properties of a drug candidate’s ADMET profile are used to determine the pharmacokinetic profile analysis of the drug candidate. In the early phase of drug development, ADMET analysis is very helpful in facilitating a considerable decrease in unsuccessful clinical trials [40]. The ADMET method was used for the lead compounds for examination. Important absorption characteristics in drug research include aqueous solubility, gastrointestinal (GI) absorption, skin permeability, and Caco2 permeability [41]. Compound **4a** had the most significant absorption percentage of 98.292 percent, followed by compound 6a with 97.555 percent and compound 6b with 96.217 percent, all of which had excellent absorbance rates (Supplementary Information, Table S2). A value of skin permeability larger than −2.5 cm/h is poor; nonetheless, all the therapeutic compounds demonstrated excellent skin permeability. Caco2 permeability was minimal (less than 0.9 cm/s) in all potential treatment candidates. Another crucial part of the ADMET research was predicting whether a P-glycoprotein may serve as a non-substrate candidate. It was discovered that each chemical acted as a substrate for the P-glycoprotein (see Supplementary Information, Table S2.

Many researchers [42] looked at the permeability of membranes in the VDSS, the CNS, and the BBB to investigate how drugs are distributed throughout the body. The log VDss that fell from −0.546 to 0.503 was considered relatively high. Regarding the permeability of the BBB membrane, log BB values ranging from −1.136 to 0.756 suggested that the drug molecules could pass across the barrier. On the other hand, the range of CNS permeability values between −2.182 and −0.767 suggested impenetrability for the central nervous system (CNS). Because of this, it was hypothesized that none of the medication candidates would be able to enter the central nervous system or pass across the blood–brain barrier (Supplementary Information, Table S2).

In the drug metabolism process in the liver, CYP450 plays a crucial role [43]. According to the results of the metabolism tests, none of the medication compounds were affected or inhibited CYP2D6 enzymes. Furthermore, drug compounds 4b and 4c did not function as inhibitors for CYP2C19 and CYP2C9 enzymes. Therefore, when measuring the total drug clearance, it is necessary to consider both hepatic and renal clearance. Furthermore, using the medication’s elimination rate, the total clearance may be used to describe the drug concentration in the body [44]. According to the forecasted findings, the excretion rates of the potential medication candidates vary from −0.142 to 2.548 mL/min/kg (Supplementary Information, Table S2).

Regarding drug development, toxicity is an important parameter that plays a big part in picking the ideal candidates for new drugs [35,36]. Except for compound **4a**, none of the other medication compounds in this study showed any signs of causing allergic skin reactions or hepatotoxic effects (Supplementary Information, Table S2). hERG inhibition (both I and II) is a critical component of the toxicity assessment process and is associated with cardiotoxicity. Inhibitory effects on hERG I inhibitors could not be seen from the substances tested. On the other hand, inhibitory effects on hERG II inhibitors could be seen from **4a**, **4b**, **4c**, and **6b** of the tested substances. In addition, only **4a** and **4b** of the potential drugs have shown toxicity for AMES or Tetrahymena Pyriformis. The toxicity analysis server made predictions about the medication candidates’ LD50, lowest observed adverse effect level (LOAEL), and maximum tolerated dose range, and the scores it came up with are shown in the Supplementary Information, Table S2. Based on these findings, the current research concluded that these bioactive drug candidates could be employed as medications that inhibit proteases responsible for COVID-19.

### 3.5. Pharmacophore Analysis

The pharmacophore model was developed by aligning the structures of the ten approved FDA active compounds against COVID-19 (training set), which were very well aligned and are present in Supplementary Information, Figures S1 and S2 [45,46].

Supplementary Information, Figure S3, shows the pharmacophore model derived thereof. Three necessary features describe it. Mainly F1: Hyd/Aro: Hydrophobic, F2: Hyd/Aro: Hydrophobic, and F3: ML/Acc/Don: Metal Ligator.

**Figure 6:**
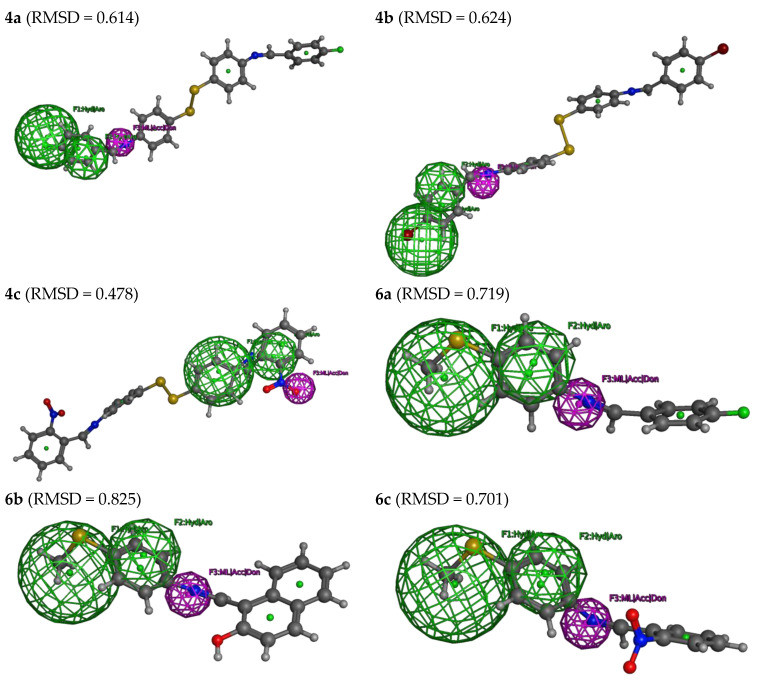
The selected hits from the pharmacophoric features for the tested compounds (**4a**, **4b**, **4c**, **6a**, **6b**, and **6c**), with their root, mean square deviation (RMSD).

As described, the three features of this model were used to search in part of the tested database (**4a**, **4b**, **4c, 6a**, **6b**, and **6c**) to identify an active COVID-19 inhibitor. However, by applying all three features of the developed pharmacophore model, none of the tested compounds (**4a**, **4b**, **4c, 6a**, **6b**, and **6c**) were excluded from further observations (Figure 6). By applying all three features of the developed pharmacophore model, it was possible to identify all the database compounds (**4a**, **4b**, **4c, 6a**, **6b**, and **6c**). Thus, all the tested compounds seem to have appropriate structures for enzyme inhibitors and to be good COVID-19 inhibitors. The deviation from the pharmacophore model was expressed as a root mean square deviation (RMSD) with superimposition on the pharmacophore model (Figure 6). According to the RMSD, the tested compounds’ reactivity can be ordered as **4c** > **4a** ≈ **4b** > **6c** ≈ **6a** > **6b**.

### 3.6. Molecular Docking

To determine the pharmacological efficacy of new compounds, researchers typically examine the degree to which new compounds are sensitive to interactions with primary targets (proteins) [47,48]. Therefore, this investigation used a methodology to explore the interaction between the chemicals of interest and the focus protein.

Predictions of the drugs’ biological activity are now being made using molecular docking, which also finds the best orientation of the ligand when it binds to the site’s pocket on the targeted protein. These predictions can now be made using molecular docking [49].

In this work, the understudy chemicals were docked to the main protease (6LU7) protein to evaluate their potential as candidates for antiviral treatment [47]. Molecular docking studies allow the prediction of the highest binding affinities because of the virtual compound screening and scoring functions used in the research. This method investigates how two molecules, a substrate and the active site binding of the target receptor, fit together like jigsaw pieces in three dimensions.

**Figure 7:**
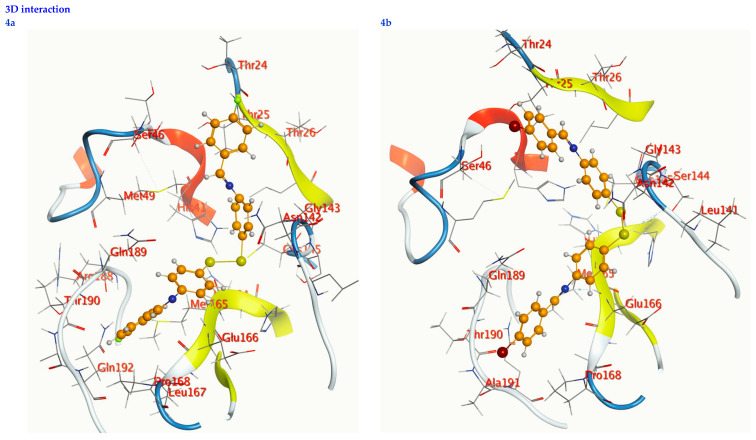

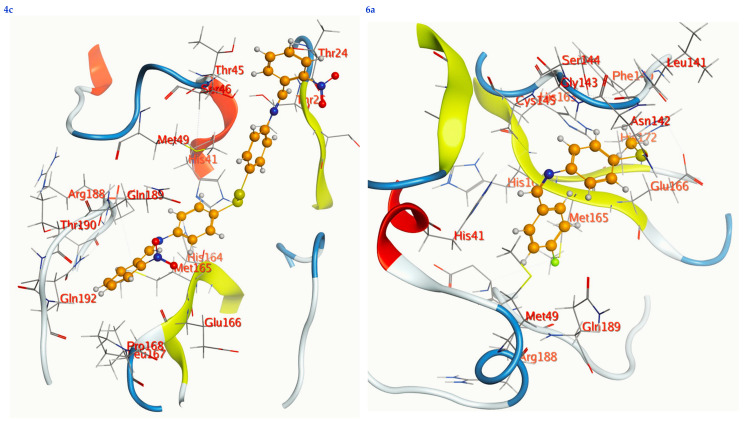

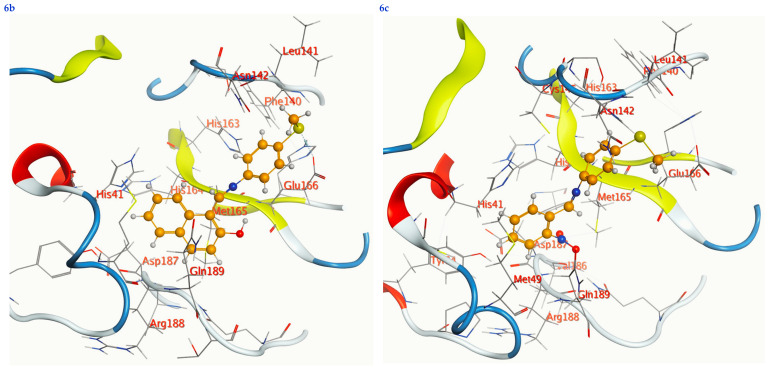

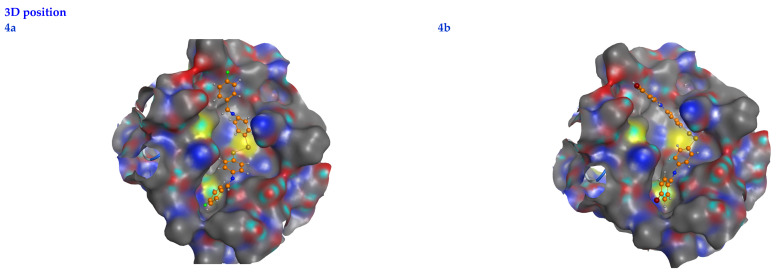

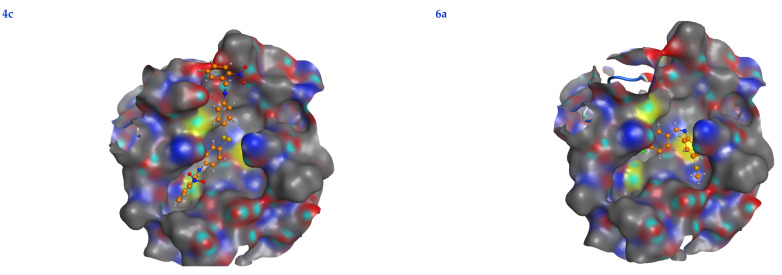

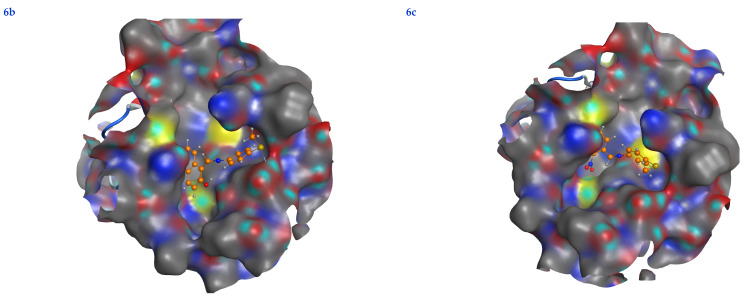

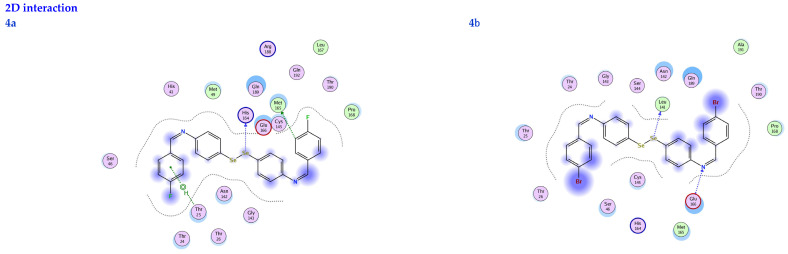

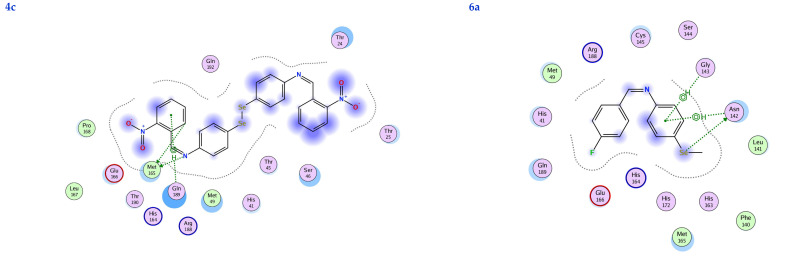

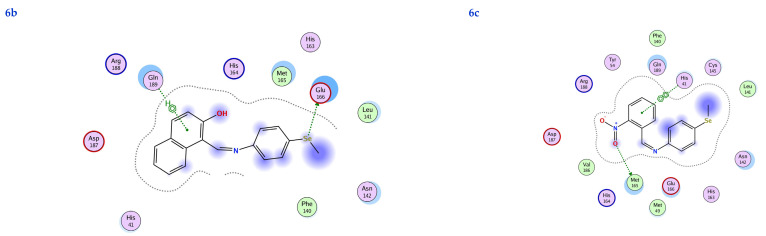
2D interactions, 3D interactions, and 3D position of the titled compounds (**4a**, **4b**, **4c**, **6a**, **6b**, and **6c**) inside the active site of the target 6LU7 receptor.

In this scenario, the 6LU7 protein is the target receptor, while the listed compounds are the substrate. Table 2 shows the molecular docking results, and Figure 7 displays the position of the optimum conformation of the studied substrates inside the binding pocket. Table 2 presents the results of the molecular docking. 

The subject substrates have significant negative docking scores (S), as shown in Figure 7 and Table 2. They link with the 6LU7 pocket in several ways, including via hydrogen bonds and hydrophobic contacts. This demonstrates a strong interaction between the docked substrates and the receptor’s active site. The sequence of the levels of inhibitory activity was as follows: **4c** > **4a** > **4b** > **6c** > **6a** > **6b**. Interestingly, the most effective compounds in the docking, **4c**, **4a**, and **4b**, were successfully incorporated into variable hydrogen bond interactions with (MET 165, GLN 189), (HIS 164, THR 25, MET 165), and (LEU 141 and GLU 166), respectively, to form a strong interaction with the substrate binding pocket of 6LU7 (Figure 7 and Table 2).

Table 2 shows that the compounds in question had high docking scores (S, Kcal/mol) and low RMSD values concerning the 6LU7. These values ranged from −8.19 Kcal/mol to 1.35 for **4c** to −6.10 Kcal/mol to 1.73 for **6b**. As a result, compound **4c** seems to be the most energetic contender, given its high docking score (−8.19 Kcal/mol) and low RMSD (1.35). In addition, compound **4c** found one pi-H interaction between the 6-ring’s connection with GLN 189 and two strong hydrogen bond interactions (N 15 with MET 165 and C 19 with MET 165).

The inhibition constant (Ki value) is a significant parameter to predict whether the synthesized molecule acts as a hit, lead, or drug candidate. Usually, a high potency is implied by a low Ki value, and it should be in the micromolar (µM) range for a molecule to be eligible as a hit or lead compound. The Ki value of a drug molecule should not be exceeded by more than a ten nanomolar range. The inhibition constant (Ki value) was calculated theoretically using the following relation (Ki = 10^[Binding Energy ( BE) ÷ 1.366]^), as described in the following references [50,51,52]. A molecule must have a Ki value in the micromolar (µM) range to be regarded as a hit or a lead chemical. This is because a low Ki value typically signals a high potency. The 6LU7 domain’s Ki values of the synthesized compounds ranged from 1.01 for **4c** to 34.26 for **6b**, which indicates that all of them have the potential to be hits and leads. According to the information in the table, the synthesized chemical with the lowest Ki value may potentially be used in therapeutic applications (Table 2).

## 4. Conclusions

In this study, new OSe-based Schiff bases were synthesized and characterized. DFT calculations performed with a B3LYP/6-311 (d, p) basis set were used to assess the resulting compounds’ geometries. The HOMO-LUMO energy gap made it possible to calculate the characteristics of molecules linked to their reactivity. Using molecular docking against the COVID-19 receptor, the nature of the interactions between the new compounds and the virus was studied. High binding energy scores were observed between the COVID-19 primary protease and OSe-based Schiff bases’ ligands (**4c** and **4a**: −8.19 and −7.33 Kcal/mol; **6b** and **6a**: −6.10 and −6.20 Kcal/mol). Our findings showed that Schiff bases containing OSe manifested promise as potential medicines for the COVID-19 pandemic. Using docking analysis, we found that 4c and 4a are potent inhibitors of the SARS-CoV-2 6LU7 protein. Lipinski’s rule and ADMET characteristics, among others, were also examined as part of the drug-likeness investigations. The ADMET findings of the OSe-based Schiff bases show that they have potent pharmacokinetic properties, including good absorption and acceptable metabolic transformation, while being shown to be safe, allowing them to be approved as trustworthy inhibitors of SARS-CoV-2.

## Figures and Tables

**Table 1:** Calculated electronic parameters (ionization potential (IP), electron affinity (EA), energy gap (ΔE), electronegativity (χ), chemical potential (cp), chemical hardness (η), softness (σ), electrophilicity index (ω), and nucleophilicity index (Nu)) of the tested compounds (**4a**, **4b**, **4c**, **6a**, **6b**, and **6c**) with atom labels using DFT/B3LYP with a 6-311G (d, p) basis set.

	E_HOMO_ (eV)	E_LUMO_ (eV)	∆E (eV)	IP (eV)	EA (eV)	χ (eV)	CP (eV)	η (eV)	σ (eV^−1^)	ω (eV)	Nu (eV^−1^)
**4a**	−5.18	−2.86	2.32	5.18	2.86	4.02	−4.02	1.16	0.43	6.97	0.14
**4b**	−4.33	−1.90	2.43	4.33	1.90	3.12	−3.12	1.22	0.41	3.99	0.25
**4c**	−4.23	−2.55	1.68	4.23	2.55	3.39	−3.39	0.84	0.59	6.84	0.15
**6a**	−5.69	−2.10	3.59	5.69	2.10	3.89	−3.89	1.79	0.28	4.23	0.24
**6b**	−5.50	−1.46	4.04	5.50	1.46	3.48	−3.48	2.02	0.25	3.00	0.33
**6c**	−5.18	−2.09	3.09	5.18	2.09	3.64	−3.64	1.55	0.32	4.28	0.23

**Table 2:** Molecular docking data, interaction type, and distance (d) between ligand and receptor of the synthesized compounds (**4a**, **4b**, **4c**, **6a**, **6b**, and **6c**).

	Ligand	Receptor	Interaction	Distance	E (kcal/mol)	S (kcal/mol)	RMSD	Ki (μM)
**4a**	Se 7	HIS 164	H-donor	2.82	−1.04	−7.33	1.37	4.33
	C 29	MET 165	H-donor	2.86	−0.80			
	6-ring	THR 25	pi-H	3.38	−1.00			
**4b**	Se 7	LEU 141	H-donor	2.78	−1.00	−7.21	1.46	5.29
	N 24	GLU 166	H-acceptor	2.83	−0.80			
**4c**	N 15	MET 165	H-donor	2.96	−0.10	−8.19	1.35	1.01
	C 19	MET 165	H-donor	3.17	−0.80			
	6-ring	GLN 189	pi-H	3.51	−0.60			
**6a**	Se 1	ASN 142	H-donor	2.72	−1.50	−6.20	1.85	28.90
	6-ring	ASN 142	pi-H	2.90	−1.20			
	6-ring	GLY 143	pi-H	3.29	−0.70			
**6b**	Se 1	GLU 166	H-donor	3.26	−4.10	−6.10	1.73	34.26
	6-ring	GLN 189	pi-H	3.69	−0.70			
**6c**	O 19	MET 165	H-donor	2.59	−0.20	−6.58	1.55	15.12
	6-ring	HIS 41	pi-pi	2.99	−0.87			

## Data Availability

The raw/processed data generated in this work are available upon request from the corresponding author.

## Supplementary Materials

The following supporting Information can be downloaded at: https://www.mdpi.com/article/10.3390/life13040912/s1, Figure S1: Structures of training set compounds; Figure S2: Alignment of ten approved FDA drugs; Figure S3: The developed pharmacophore model; Table S1: Drug Likeness parameters; Table S2: ADMET properties using pkCSM web server; Copies of the ^1^H-NMR & ^13^C-NMR, IR, and MS spectra of the organoselenium compounds.

## References

[1] Shaaban S., El-Lateef H.M.A., Khalaf M.M., Gouda M., Youssef I. (2022). One-Pot Multicomponent Polymerization, Metal-, and Non-Metal-Catalyzed Synthesis of Organoselenium Compounds. Polymers.

[2] Chuai H., Zhang S.-Q., Bai H., Li J., Wang Y., Sun J., Wen E., Zhang J., Xin M. (2021). Small molecule selenium-containing compounds: Recent development and therapeutic applications. Eur. J. Med. Chem..

[3] Kalimuthu K., Keerthana C.K., Mohan M., Arivalagan J., Christyraj J.R.S.S., Firer M.A., Choudry M.H.A., Anto R.J., Lee Y.J. (2021). The emerging role of selenium metabolic pathways in cancer: New therapeutic targets for cancer. J. Cell. Biochem..

[4] Xu J., Gong Y., Sun Y., Cai J., Liu Q., Bao J., Yang J., Zhang Z. (2020). Impact of Selenium Deficiency on Inflammation, Oxidative Stress, and Phagocytosis in Mouse Macrophages. Biol. Trace Elem. Res..

[5] Nogueira C.W., Barbosa N.V., Rocha J.B.T. (2021). Toxicology and pharmacology of synthetic organoselenium compounds: An update. Arch. Toxicol..

[6] Nogueira C.W., Rocha J.B.T. (2011). Toxicology and pharmacology of selenium: Emphasis on synthetic organoselenium compounds. Arch. Toxicol..

[7] Makhal P.N., Nandi A., Kaki V.R. (2021). Insights into the recent synthetic advances of organoselenium compounds. ChemistrySelect.

[8] Lenardão E.J., Santi C., Sancineto L. (2018). New Frontiers in Organoselenium Compounds.

[9] Phadnis P.P. (2021). Synthesis Strategies for Organoselenium Compounds and Their Potential Applications in Human Life. Handbook on Synthesis Strategies for Advanced Materials.

[10] Zhao X., Liao L. (2021). Modern Organoselenium Catalysis: Opportunities and Challenges. Synlett.

[11] Benelli J.L., Poester V.R., Munhoz L.S., Melo A.M., Trápaga M.R., A Stevens D., Xavier M.O. (2021). Ebselen and diphenyl diselenide against fungal pathogens: A systematic review. Med. Mycol..

[12] Jin Z., Du X., Xu Y., Deng Y., Liu M., Zhao Y., Zhang B., Li X., Zhang L., Peng C. (2020). Structure of Mpro from SARS-CoV-2 and discovery of its inhibitors. Nature.

[13] Mansour M.A., AboulMagd A.M., Abdel-Rahman H.M. (2020). Quinazoline-Schiff base conjugates: In silico study and ADMET predictions as multi-target inhibitors of coronavirus (SARS-CoV-2) proteins. RSC Adv..

[14] Alshammari M.B., Ramadan M., Aly A.A., El-Sheref E.M., Bakht A., Ibrahim M.A., Shawky A.M. (2020). Synthesis of potentially new schiff bases of N-substituted-2-quinolonylacetohydrazides as anti-COVID-19 agents. J. Mol. Struct..

[15] Shaaban S., Adam M.S.S., El-Metwaly N.M. (2022). Novel organoselenium-based N-mealanilic acid and its zinc (II) chelate: Catalytic, anticancer, antimicrobial, antioxidant, and computational assessments. J. Mol. Liq..

[16] Kohn W., Sham L.J. (1965). Self-consistent equations including exchange and correlation effects. Phys. Rev..

[17] Kuntz I.D., Blaney J.M., Oatley S.J., Langridge R., Ferrin T.E. (1982). A geometric approach to macromolecule-ligand interactions. J. Mol. Biol..

[18] Ma Y.T., Liu M.C., Zhou Y.B., Wu H.Y. (2021). Synthesis of Organoselenium Compounds with Elemental Selenium. Adv. Synth. Catal..

[19] Al-Gaber M.A.I., El-Lateef H.M.A., Khalaf M.M., Shaaban S., Shawky M., Mohamed G.G., Abdou A., Gouda M., Abu-Dief A.M. (2023). Design, Synthesis, Spectroscopic Inspection, DFT and Molecular Docking Study of Metal Chelates Incorporating Azo Dye Ligand for Biological Evaluation. Materials.

[20] Shokr E.K., Kamel M.S., Abdel-Ghany H., Remaily M.A.E.A.A.A.E., Abdou A. (2022). Synthesis, characterization, and DFT study of linear and non-linear optical properties of some novel thieno[2,3-b]thiophene azo dye derivatives. Mater. Chem. Phys..

[21] Elkanzi N.A.A., Ali A.M., Albqmi M., Abdou A. (2022). New Benzimidazole-Based Fe (III) and Cr (III) Complexes: Characterization, Bioactivity Screening, and Theoretical Implementations Using DFT and Molecular Docking Analysis. Appl. Organomet. Chem..

[22] Fukui K., Yonezawa T., Shingu H. (1952). A Molecular Orbital Theory of Reactivity in Aromatic Hydrocarbons. J. Chem. Phys..

[23] Fukui K., Yonezawa T., Nagata C., Shingu H. (1954). Molecular Orbital Theory of Orientation in Aromatic, Heteroaromatic, and Other Conjugated Molecules. J. Chem. Phys..

[24] Al-Wabli R.I., Resmi K., Mary Y.S., Panicker C.Y., Attia M.I., El-Emam A.A., Van Alsenoy C. (2016). Vibrational spectroscopic studies, Fukui functions, HOMO-LUMO, NLO, NBO analysis and molecular docking study of (E)-1-(1,3-benzodioxol-5-yl)-4,4-dimethylpent-1-en-3-one, a potential precursor to bioactive agents. J. Mol. Struct..

[25] Missioui M., Said M.A., Demirtaş G., Mague J.T., Al-Sulami A., Al-Kaff N.S., Ramli Y. (2021). A possible potential COVID-19 drug candidate: Diethyl 2-(2-(2-(3-methyl-2-oxoquinoxalin-1(2H)-yl)acetyl)hydrazono)malonate: Docking of disordered independent molecules of a novel crystal structure, HSA/DFT/XRD and cytotoxicity. Arab. J. Chem..

[26] Alghuwainem Y.A.A., El-Lateef H.M.A., Khalaf M.M., Amer A.A., Abdelhamid A.A., Alzharani A.A., Alfarsi A., Shaaban S., Gouda M., Abdou A. (2022). Synthesis, DFT, Biological and Molecular Docking Analysis of Novel Manganese(II), Iron(III), Cobalt(II), Nickel(II), and Copper(II) Chelate Complexes Ligated by 1-(4-Nitrophenylazo)-2-naphthol. Int. J. Mol. Sci..

[27] Chaudhary A.P., Bharti S.K., Kumar S., Ved K., Padam K. (2017). Study of molecular structure, chemical reactiv-ity and first hyperpolarizability of a newly synthesized N-(4-oxo-2-phenylquinazolin-3 (4H)-yl)-1H-indole-2-carboxamide using spectral analysis. J. Mol. Struct..

[28] Abdou A., Mostafa H.M., Abdel-Mawgoud A.-M.M. (2022). Seven metal-based bi-dentate NO azocoumarine complexes: Synthesis, physicochemical properties, DFT calculations, drug-likeness, in vitro antimicrobial screening and molecular docking analysis. Inorg. Chim. Acta.

[29] Pearson R.G. (1963). Hard and Soft Acids and Bases. J. Am. Chem. Soc..

[30] Elkanzi N.A., Hrichi H., Salah H., Albqmi M., Ali A.M., Abdou A. (2023). Synthesis, physicochemical properties, biological, molecular docking and DFT investigation of Fe(III), Co(II), Ni(II), Cu(II) and Zn(II) complexes of the 4-[(5-oxo-4,5-dihydro-1,3-thiazol-2-yl)hydrazono]methyl}phenyl 4-methylbenzenesulfonate Schiff-base ligand. Polyhedron.

[31] Rajamanickam R., Mannangatty R., Sampathkumar J., Senthamaraikannan K., Diravidamani B. (2022). Synthesis, crystal structure, DFT and molecular docking studies of N-acetyl-2,4-[diaryl-3-azabicyclo[3.3.1]nonan-9-yl]-9-spiro-4′-acetyl-2′-(acetylamino)-4′,9-dihydro-[1′,3′,4′]-thiadiazoles: A potential SARS-nCoV-2 Mpro (COVID-19) inhibitor. J. Mol. Struct..

[32] Alghuwainem Y.A., El-Lateef H.M.A., Khalaf M.M., Abdelhamid A.A., Alfarsi A., Gouda M., Abdelbaset M., Abdou A. (2023). Synthesis, structural, DFT, antibacterial, antifungal, anti-inflammatory, and molecular docking analysis of new VO(II), Fe(III), Mn(II), Zn(II), and Ag(I) complexes based on 4-((2-hydroxy-1-naphthyl)azo) benzenesulfonamide. J. Mol. Liq..

[33] Lipinski C.A., Lombardo F., Dominy B.W., Feeney P.J. (2001). Experimental and computational approaches to estimate solubility and permeability in drug discovery and development settings. Adv. Drug Deliv. Rev..

[34] Owens J. (2003). Chris Lipinski discusses life and chemistry after the Rule of Five. Drug Discov. Today.

[35] Lin J.H., Borchardt R., Kerns E., Lipinski C., Thakker D., Wang B. (2006). Challenges in drug discovery: Lead optimization and prediction of human pharmacokinetics. Pharmaceutical Profiling in Drug Discovery for Lead Selection.

[36] Martin Y.C. (2005). A Bioavailability Score. J. Med. Chem..

[37] Leeson P.D., Bento A.P., Gaulton A., Hersey A., Manners E.J., Radoux C.J., Leach A.R. (2021). Target-Based Evaluation of “Drug-like” Properties and Ligand Efficiencies. J. Med. Chem..

[38] Daina A., Michielin O., Zoete V. (2017). SwissADME: A free web tool to evaluate pharmacokinetics, drug-likeness and medicinal chemistry friendliness of small molecules. Sci. Rep..

[39] Abdullahi S.H., Uzairu A., Shallangwa G.A., Uba S., Umar A.B. (2022). Computational modeling, ligand-based drug design, drug-likeness and ADMET properties studies of series of chromen-2-ones analogues as anti-cancer agents. Bull. Natl. Res. Cent..

[40] Zoete V., Daina A., Bovigny C., Michielin O. (2016). SwissSimilarity: A Web Tool for Low to Ultra High Throughput Ligand-Based Virtual Screening. J. Chem. Inf. Model..

[41] Pires D.E.V., Blundell T.L., Ascher D.B. (2015). pkCSM: Predicting Small-Molecule Pharmacokinetic and Toxicity Properties Using Graph-Based Signatures. J. Med. Chem..

[42] Han L., Pang K., Fu T., Phillips C.J.C., Gao T. (2020). Nano-selenium Supplementation Increases Selenoprotein (Sel) Gene Expression Profiles and Milk Selenium Concentration in Lactating Dairy Cows. Biol. Trace Elem. Res..

[43] Cortes C., Vapnik V. (1995). Support-vector networks. Mach. Learn.

[44] Balaramnavar V.M., Ahmad K., Saeed M., Ahmad I., Kamal M., Jawed T. (2020). Pharmacophore-based approaches in the rational repurposing technique for FDA approved drugs targeting SARS-CoV-2 Mpro. RSC Adv..

[45] Maji S., Pattanayak S.K., Sen A., Badavath V.N., Rudrapal M., Egbuna C. (2022). Chapter 6—Pharmacophore modeling in drug design. Drug Discovery Update, Computer Aided Drug Design (CADD): From Ligand-Based Methods to Structure-Based Approaches.

[46] Sahu S.N., Satpathy S.S., Pattnaik S., Mohanty C., Pattanayak S.K. (2022). Boerhavia diffusa plant extract can be a new potent therapeutics against mutant nephrin protein responsible for type1 nephrotic syndrome: Insight into hydrate-ligand docking interactions and molecular dynamics simulation study. J. Indian Chem. Soc..

[47] Mengist H.M., Fan X., Jin T. (2020). Designing of improved drugs for COVID-19: Crystal structure of SARS-CoV-2 main protease Mpro. Signal Transduct. Target. Ther..

[48] Moharana M., Pattanayak S.K. (2023). Molecular recognition of bio-active triterpenoids from Swertia chirayita towards hepatitis Delta antigen: A mechanism through docking, dynamics simulation, Gibbs free energy landscape. J. Biomol. Struct. Dyn..

[49] Al-Khafaji K., Tok T.T. (2020). Molecular dynamics simulation, free energy landscape and binding free energy computations in exploration the anti-invasive activity of amygdalin against metastasis. Comput. Methods Programs Biomed..

[50] Al-Abdallah B., Al-Faiyz Y.S., Shaaban S. (2022). Organoselenocyanates Tethered Methyl Anthranilate Hybrids with Promising Anticancer, Antimicrobial, and Antioxidant Activities. Inorganics.

[51] Al-Abdallah B., Al-Faiyz Y.S., Shaaban S. (2022). Anticancer, Antimicrobial, and Antioxidant Activities of Or-ganodiselenide-Tethered Methyl Anthranilates. Biomolecules.

[52] Abu-Dief A.M., Alotaibi N.H., Al-Farraj E.S., Qasem H.A., Alzahrani S., Mahfouz M.K., Abdou A. (2022). Fabrication, structural elucidation, theoretical, TD-DFT, vibrational calculation and molecular docking studies of some novel adenine imine chelates for biomedical applications. J. Mol. Liq..

