# Supplementary material for: Synthesis and in Silico Investigation of Organoselenium-Clubbed Schiff Bases as Potential Mpro Inhibitors for the SARS-CoV-2 Replication
Source: Life (Basel). 2023 Mar 30;13(4):912. doi: 10.3390/life13040912 (PMC10141725; doi:10.3390/life13040912)
Supplement: Supplementary file 1 [file life-13-00912-s001.zip › life-2277576-supplementary.pdf]

Supplementary Information

Synthesis and in Silico Investigation of Organoselenium Clubbed Schiff Bases as Potential M^{pro} Inhibitors for the SARS-CoV-2 Replication

Saad Shaaban [1,2,*], Aly Abdou [3,*], Abdulrahman G. Alhamzani [4], Mortaga M. Abou-Krisha [4,5], Mahmoud A. Al-Qudah [4,6], Mohamed Alaasar [7,8], Ibrahim Youssef [2] and Tarek A. Yousef [4,9,*]

[1] Department of Chemistry, College of Science, King Faisal University, Al-Ahsa 31982, Saudi Arabia
[2] Department of Chemistry, Faculty of Science, Mansoura University, Mansoura 35516, Egypt
[3] Department of Chemistry, Faculty of Science, Sohag University, Sohag 82524, Egypt
[4] College of Science, Chemistry Department, Imam Mohammad Ibn Saud Islamic University, Riyadh 11623, Saudi Arabia
[5] Department of Chemistry, South Valley University, Qena 83523, Egypt
[6] Department of Chemistry, Faculty of Science, Yarmouk University, Irbid 21163, Jordan
[7] Institute of Chemistry, Martin Luther University Halle-Wittenberg, 06108 Halle (Saale), Germany
[8] Department of Chemistry, Faculty of Science, Cairo University, Giza 12613, Egypt
[9] Department of Toxic and Narcotic Drug, Forensic Medicine, Mansoura Laboratory, Medicolegal Organization, Ministry of Justice, Cairo 11435, Egypt
* Correspondence: sibrahim@kfu.edu.sa or dr_saad_chem@mans.edu.eg (S.S.); aly_abdou@science.sohag.edu.eg (A.A.); tayousef@imamu.edu.sa (T.A.Y.)

Computational calculations

DFT calculations

The geometry of the study compounds was optimized with the use of the hybrid correlation functional (B3LYP) and the 6-311 (d, p) basis set, both of which were implemented in the Gaussian 09 software. The HOMO and LUMO energies were used to evaluate the following quantum chemical parameters: ionization potential (I.P.), electron affinity (E.A.), energy gap (ΔE), electronegativity (χ), chemical potential (cp), chemical hardness (η), softness (σ), electrophilicity index (ω), and nucleophilicity index (Nu).

Pharmacophore investigation

Here, a database, as training set, was created from ten approved FDA drugs used in the treatment of COVID-19; Chloroquine, Cycloheximide, Emetine, Exalamide, Hycanthone, Lycorine, Promazin, Propranalol, Trilorene, and Zoxazolamine, Fig. (S1). The pharmacophore generation protocol was performed using the MOE 2015. The database compounds were undergoing energy minimization, and then undergo flexible alignments, and pharmachophore search. The 'feature mapping' protocol was run to detect common features in the database.

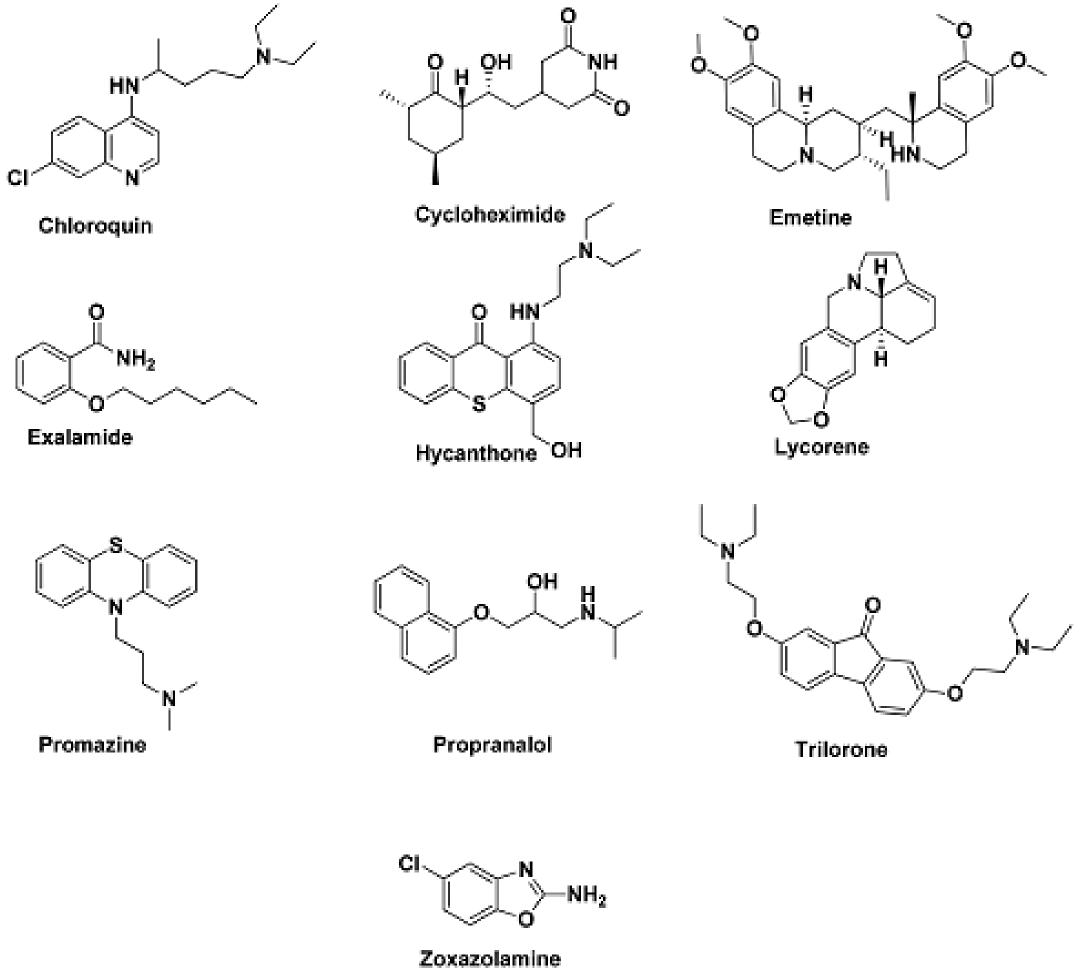

Figure S1. Structures of training set compounds.

Molecular docking investigation

The chemicals referred to in the title were put to use as ligands (substrates) so that their capacity to bind to SARS-CoV-2 protease (PDB ID: 6LU7) could be investigated using the molecular docking method. The molecular orbital energy was then utilized to establish the compounds' therapeutic efficacy. In addition to this, this was carried out in order to ascertain the effectiveness of the compounds in question as antiviral agents.

A three-dimensional model of the target compounds was generated via the builder interface of the MOE software. After that, the target compounds were made into new databases, and protonate 3D, partial charges, and energy minimization were used in the ligand synthesis process.

After that, the obtained information was saved as an MDB file and included into the docking calculation.

The X-ray crystallographic structure of the SARS-CoV-2 protease, which has the PDB ID of 6LU7, was obtained from the Protein Data Bank database, which may be accessed at http://www.rcsb.org. The hydrogen atoms and partial charges are added to the protein with the protonate 3D application included in MOE. After that, the link between the atoms and the sort of atoms were examined, hydrogen atoms were added, the receptor was selected, and the potential of its atoms was set. The MOE Alpha Site Finder was used to conduct a search for the active site in the enzyme structure. The active site was modified to accommodate the residues that were bound to the receptor. Alpha spheres were acquired, and they were then put to use in the production of fake atoms.

The docking experiments were carried out so that the internal binding free energy of the inhibitor (target receptor) could be calculated (protein). In docking research, the scoring was done using a function called the London dG scoring function. Each docking experiment was composed of one hundred separate runs, each of which tried to get the greatest possible score. Docking patterns and interaction parameters were exported so that an inhibitory activity rating could be determined using a scoring system (S, kcal/mol) and an analysis of the interaction's features could be performed.

As prospective control ligands, the binding potential of chloroquine and hydroxychloroquine has been investigated and analyzed. The energy-efficient 3D structure of the ligands was created by converting the 2D structure of the ligands.

Drug likeness and ADMET prediction

In the field of drug development, computer-based ADME evaluations are becoming more used. pkCSM and SwissADME were used to conduct ADMET (Absorption, Distribution, Metabolism, Excretion, and Toxicity) and drug-likeness prediction on drug candidate molecules. The properties of a drug's absorption (gastrointestinal absorption, bioavailability, water solubility (log S), Caco-2 and skin permeability), distribution (blood–brain barrier (BBB), central nervous system (CNS) permeability, the volume of distribution (VDss) unbound state), metabolism (various metabolic enzymes of Cytochromes P450 (CYP)), excretion (drug and renal clearance),

and toxicity (AMES, acute and chronic In addition, these toxicological forecasts have been used in the application of the Lipinski rule as well as the bioavailability scores.

Table S1. Drug Likeness parameters .

	**4a**	**4b**	**4c**	**6a**	**6b**	**6c**
**MW**	554.35 g/mol	676.16 g/mol	608.37 g/mol	292.21 g/mol	340.28 g/mol	319.22 g/mol
**log*P***	4.7402	5.987	3.2784	2.9539	3.6736	2.723
**n_{Rot}**	7	7	9	3	3	4
**nHBA**	4	2	6	2	2	3
**nHBD**	0	0	0	0	1	0
**Lipinski rule**	1violations: MW>500	1violations: MW>500	1 violation: MW>500	0 violation	0 violation	0 violation
**ABS**	0.17	0.17	0.55	0.55	0.55	0.55
**Log S**	-6.66	-7.88	-8.02	-3.77	-5.03	-4.46
**GI**	High	High	Low	High	High	High
**Pgp**	Yes	Yes	Yes	No	No	Yes
**BBB**	Yes	No	No	Yes	Yes	Yes
**TPSA**	24.72 Å²	24.72 Å²	116.36 Å²	12.36 Å²	32.59 Å²	58.18 Å²
**LogKp**	-5.17 cm/s	-5.08 cm/s	-5.89 cm/s	-5.37 cm/s	-5.10 cm/s	-5.73 cm/s
**SA**	3.53	3.63	3.91	2.69	2.96	3.02

n_{Rot}: number of rotatable bonds; **MW**: molecular weight; **nHBD**: number of hydrogen bond donors; **HBA**: number ofhydrogenbondacceptors; **log*P***:n–octanol/water partition coefficient; **S.A.**: synthetic accessibility;**BBB**: blood-brain barrier;**TPSA**: topologicalpolar surface area; **G.I.**: gastrointestinal absorption; **Pgp**: P-glycoprotein substrate; **ABS**: AbbotBioavailabilityScore;**LogKp:** skin permeation (cm/s).

Table S2.ADMET properties using pkCSM web server.

		4a	4b	4c	6a	6b	6c
**Absorption**	Water solubility	-6.711	-7.007	-5.58	-4.191	-4.9	-4.312
	Skin Permeability	-2.729	-2.732	-2.735	-1.921	-2.458	-2.533
	Intestinal absorption (human)	98.292	96.217	95.231	97.555	96.062	95.931
	Caco2 permeability	1.121	1.023	0.615	1.336	1.61	1.321
	P-glycoprotein substrate	Yes	Yes	Yes	No	Yes	Yes
	P-glycoprotein I inhibitor	Yes	Yes	Yes	No	No	No
	P-glycoprotein II inhibitor	Yes	Yes	Yes	No	No	No
**Distribution**	VDss (human)	-0.353	0.071	-0.546	0.444	0.428	0.503
	Fraction unbound (human)	0.209	0.134	0.182	0.113	0.068	0.008
	BBB permeability	0.856	0.721	-1.136	0.756	0.19	-0.186
	CNS permeability	-0.846	-0.767	-2.182	-1.371	-1.478	-2.095
**Metabolism**	CYP1A2 inhibitior	Yes	Yes	No	Yes	Yes	Yes
	CYP3A4 substrate	Yes	Yes	Yes	Yes	Yes	Yes
	CYP3A4 inhibitior	Yes	Yes	Yes	No	No	No
	CYP2D6 substrate	No	No	No	No	No	No
	CYP2D6 inhibitior	No	No	No	No	No	No
	CYP2C9 inhibitior	Yes	No	No	Yes	Yes	Yes
	CYP2C19 inhibitior	Yes	No	No	Yes	Yes	Yes
**Excretion**	Total Clearance	2.2	-0.142	2.548	1.498	1.777	1.684
	Renal OCT2 substrate	No	No	No	No	No	No
**Toxicity**	hERG I inhibitor	No	No	No	No	No	No
	hERG II inhibitor	Yes	Yes	Yes	No	Yes	No
	Max. tolerated dose (human)	0.598	0.398	0.345	0.659	0.33	0.364
	AMES toxicity	No	No	Yes	Yes	Yes	Yes
	Oral Rat Acute Toxicity (LD50)	2.992	2.737	3.078	1.956	2.121	2.33
	Oral Rat Chronic Toxicity (LOAEL)	2.435	1.013	1.687	0.757	0.467	1.378
	*T.Pyriformis*toxicity	0.293	0.301	0.286	2.053	1.213	1.76
	Hepatotoxicity	Yes	No	No	No	No	No
	Skin Sensitisation	No	No	No	No	No	No
	Minnow toxicity	-0.931	-1.681	-2.51	0.415	-0.088	-0.195

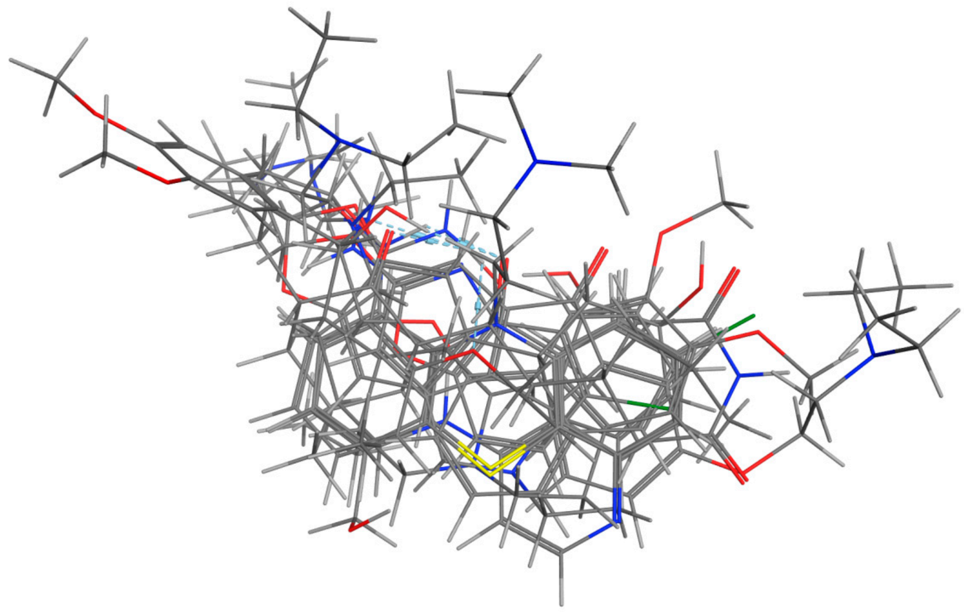

Figure S2. Alignment of ten approved FDA drugs (Chloroquine, Cycloheximide, Emetine, Exalamide, Hycanthone, Lycorine, Promazin, Propranalol, Trilorene, and Zoxazolamine) from the training set, carbon atoms are in gray, oxygen atoms are in red, nitrogen atom in blue.

(a) (b)

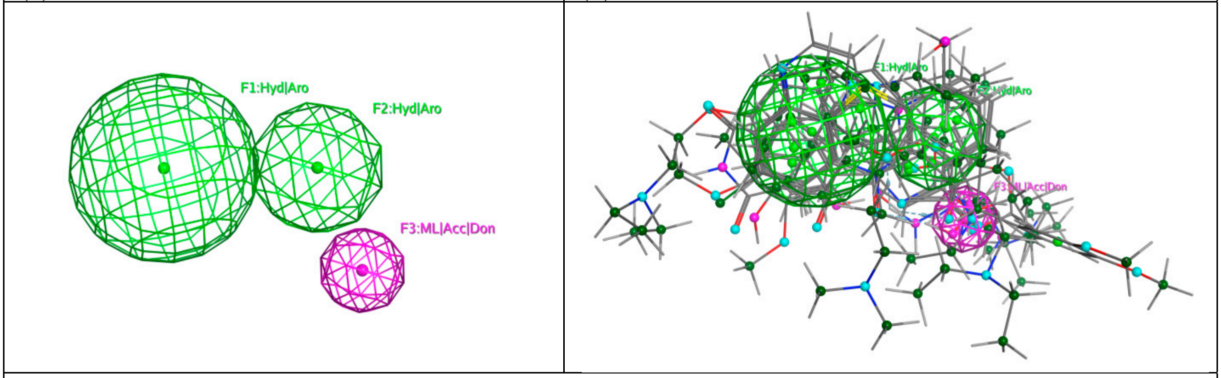

Figure S3. (a) The developed pharmacophore model, (b) Alignment of common feature pharmacophore model with the training set; Hyd: Hydrophobic, Aro: Aromatic center, Acc: H-bond acceptor, Don: H-bond acceptor, ML: Metal Ligator.

Synthesis of the organoselenium compounds

4-Selenocyanatoaniline (2), 4,4′-diselanediyldianiline (3), and 4-(methylselanyl)aniline (5) were prepared from aniline employing the modified literature method. Briefly, 4-(methylselanyl)aniline (5) was prepared in 57 % yield from 4,4′-diselanediyldianiline (3) (2 mmol), methyl iodide (4.4 mmol), NaOH (2 mol), and $NaBH_4$ (50 mmol) in MeOH (30 mL). The reaction continued at RT for approximately 2 hr. The reaction was extracted using CH_2Cl_2 and removed under vacuum to give a brown oil in 91 % yield.

4-Selenocyanatoaniline (2) was obtained as a yellow solid (88 % yield). Mp: 73–74 °C. ^{1}H NMR (400 MHz, $CDCl_3$) δ 7.44 (d, J = 8.4 Hz, 2H, Ar-H), 6.64 (d, J = 8.4 Hz, 2H, Ar-H), 3.95 (s, 2H, NH_2).

4,4′-Diselanediyldianiline (3) was obtained as pale-yellow crystals (82 % yield. Mp: 78–80 °C. ^{1}H NMR (400 MHz, $CDCl_3$) δ 7.3 (m, 4H, Ar-H), 6.5 (m, 4H, Ar-H), 3.7 ppm (s, 4H, NH_2).

4-(Methylselanyl)aniline (4) was synthesized as a brown oil (91 % yield). ^{1}H NMR (400 MHz, $CDCl_3$) δ 7.31 (d, J = 9.0 Hz, 2H, Ar-H), 6.61(d, J = 9.0 Hz, 2H, Ar-H), 3.72 (br s, 2H, NH_2), 2.66 (s, 3H, CH_3).

Copies of the ^{1}H-NMR &^{13}C-NMR, IR, and MS spectra of the organoselenium compounds

*N,N′-(diselanediylbis(4,1-phenylene))bis(1-(4-fluorophenyl)methanimine) (**4a**)*

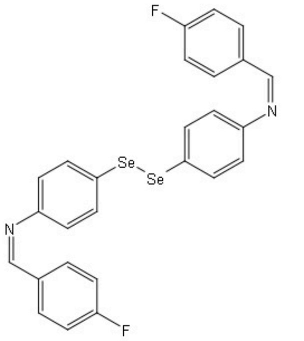

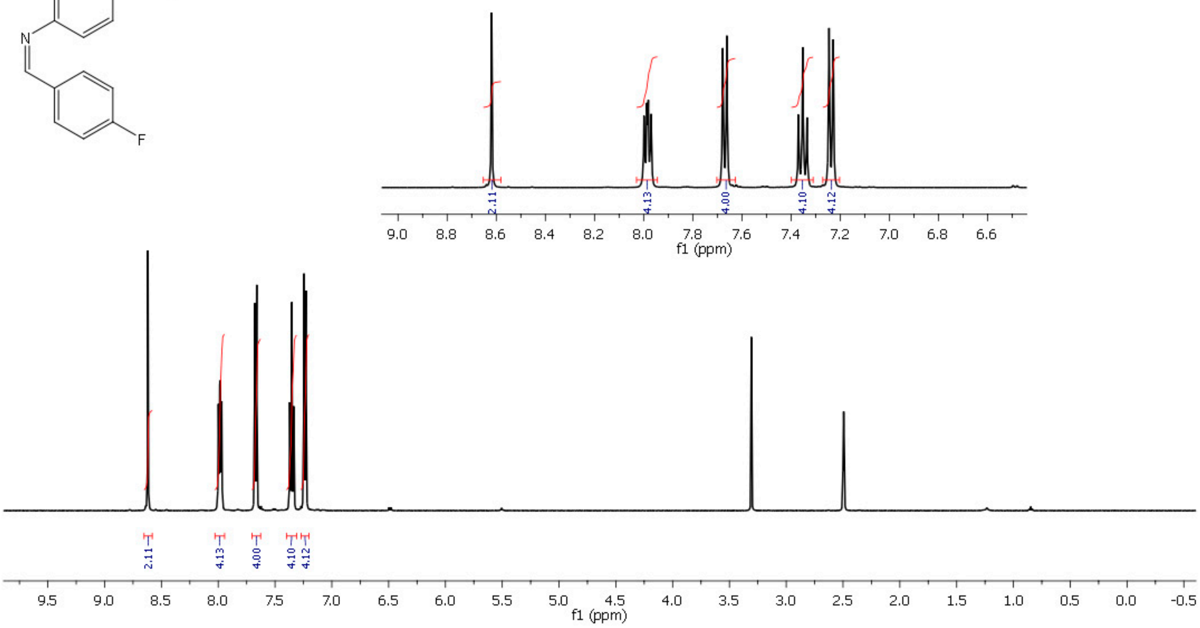

^{1}HNMR chart of compound**4a**

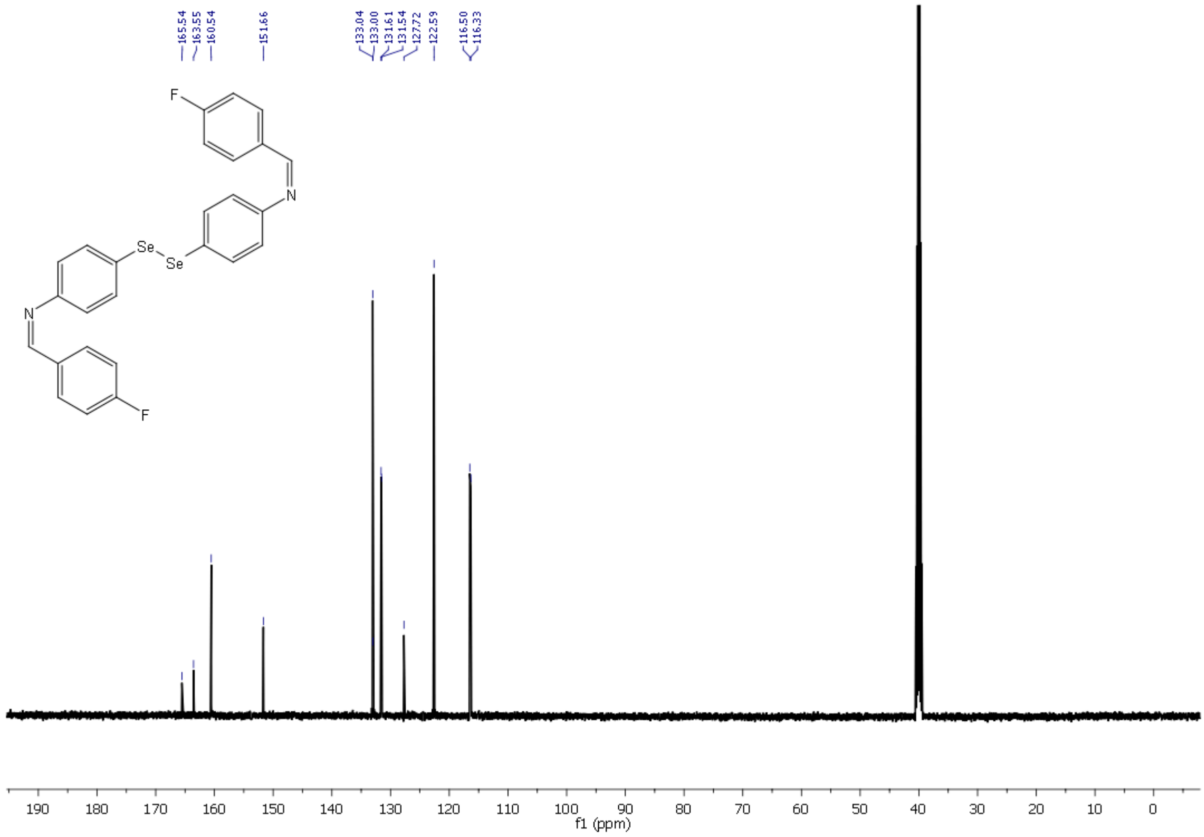

^13^CNMR chart of compound**4a**

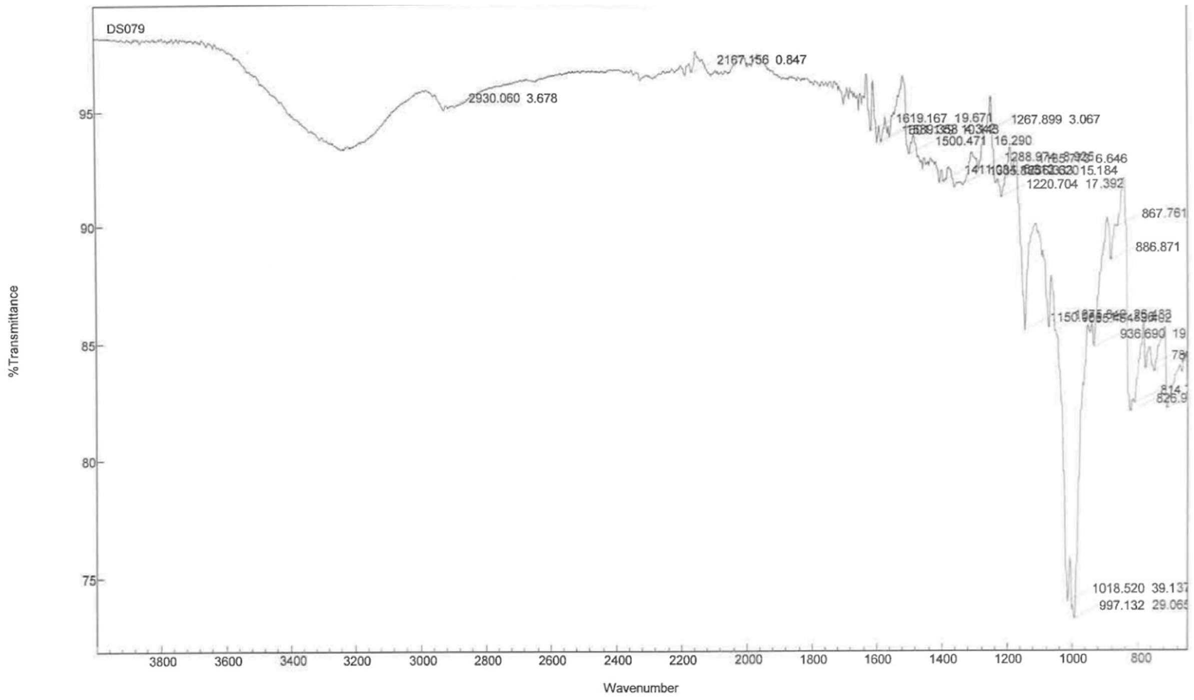

IR chart of compound **4a**

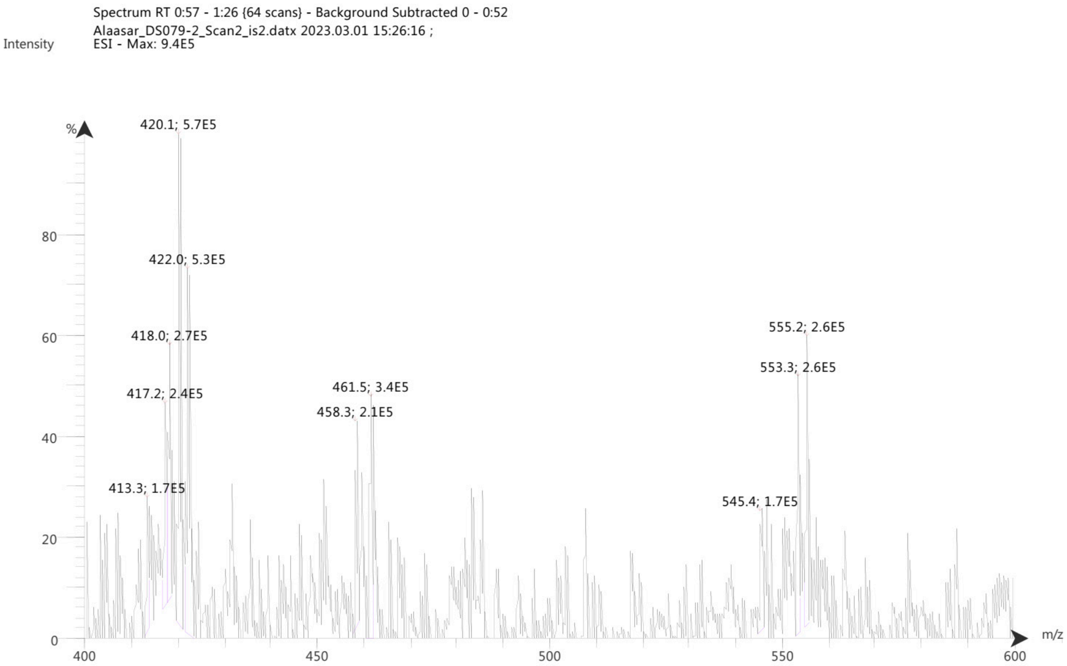

MS chart of compound **4a**

N,N'-(diselanediylbis(4,1-phenylene))bis(1-(4-bromophenyl)methanimine) (***4b***)

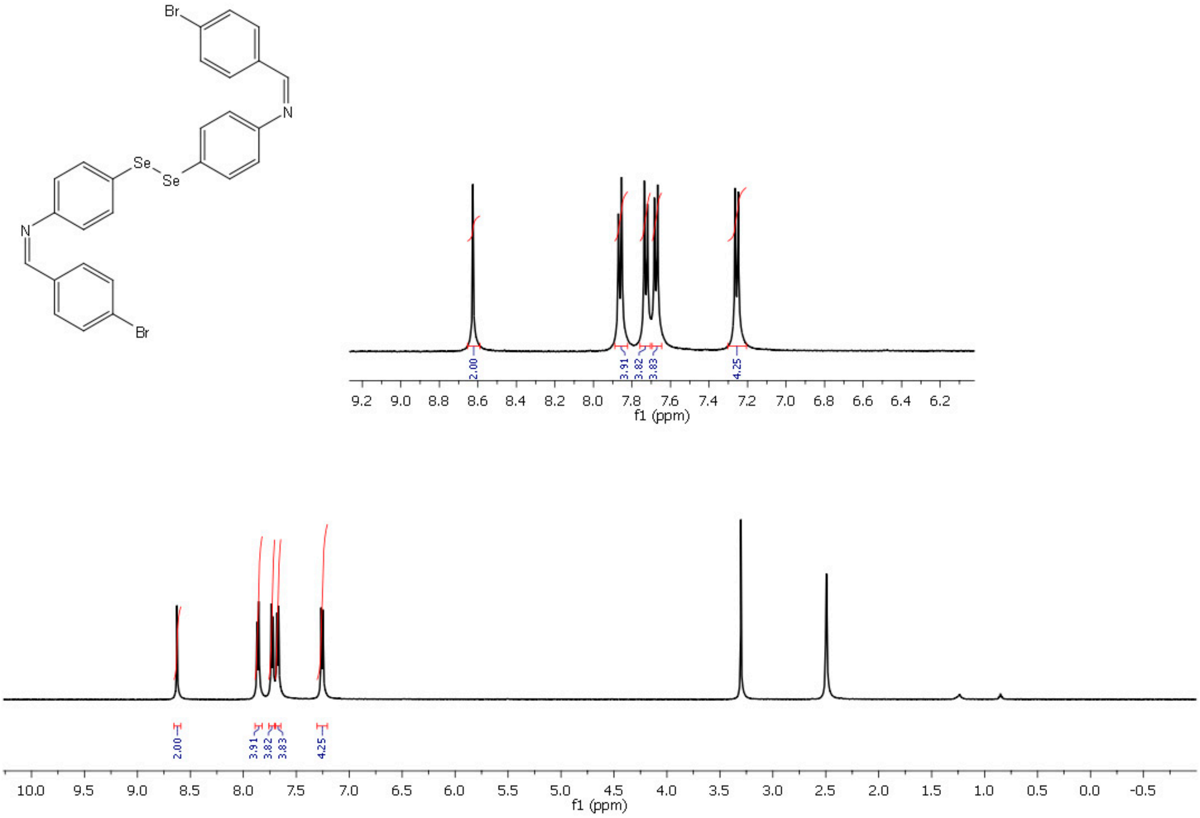

^{1}HNMR chart of compound**4b**

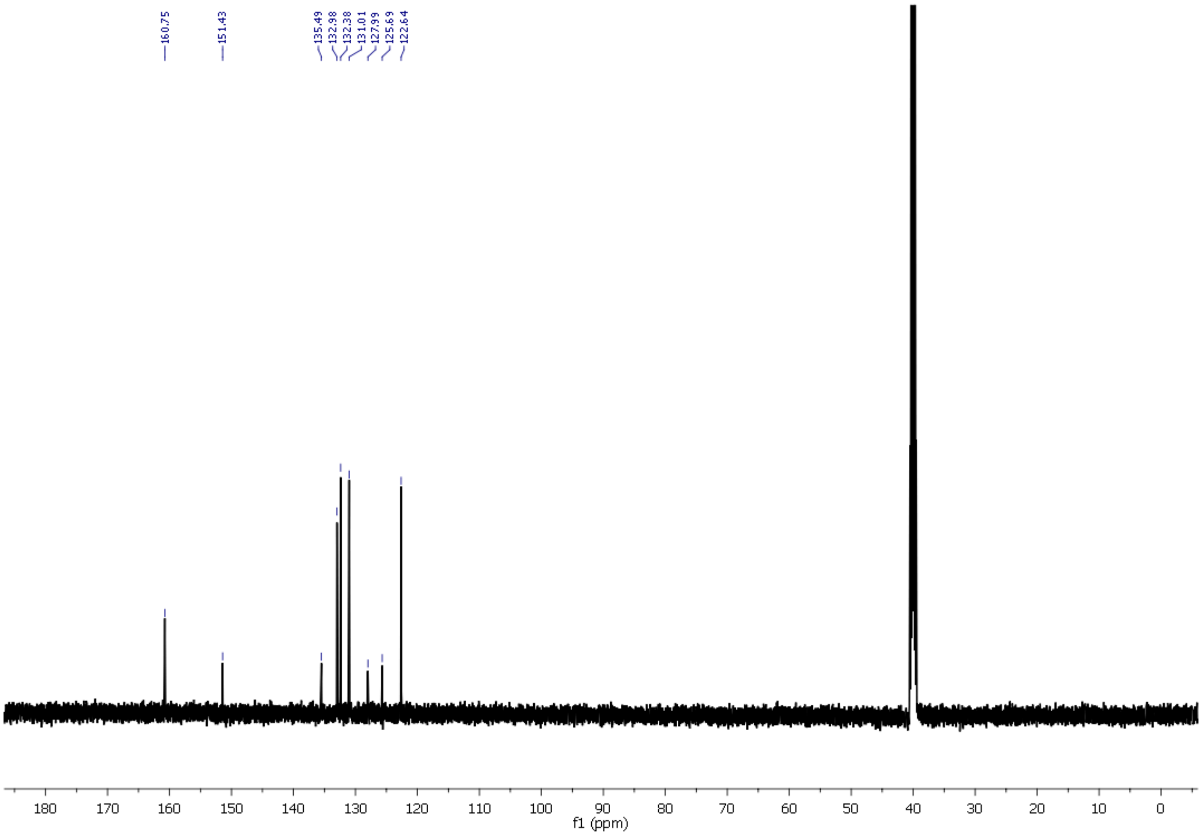

^{13}CNMR chart of compound**4b**

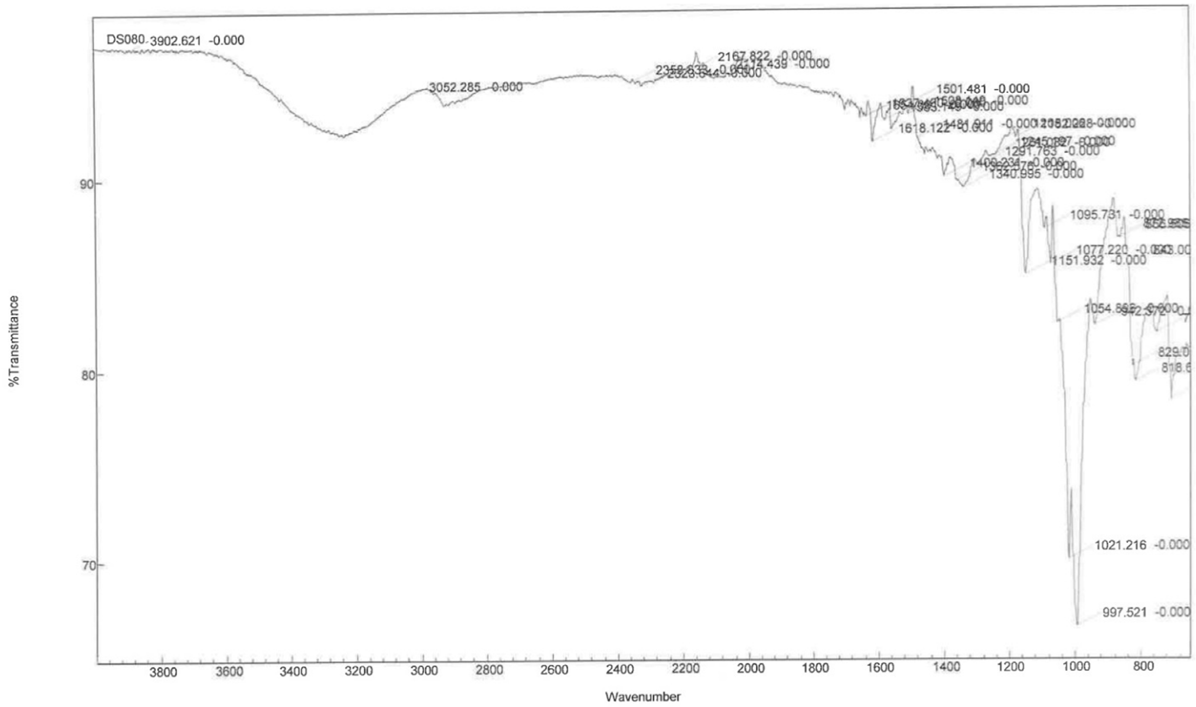

IR chart of compound**4b**

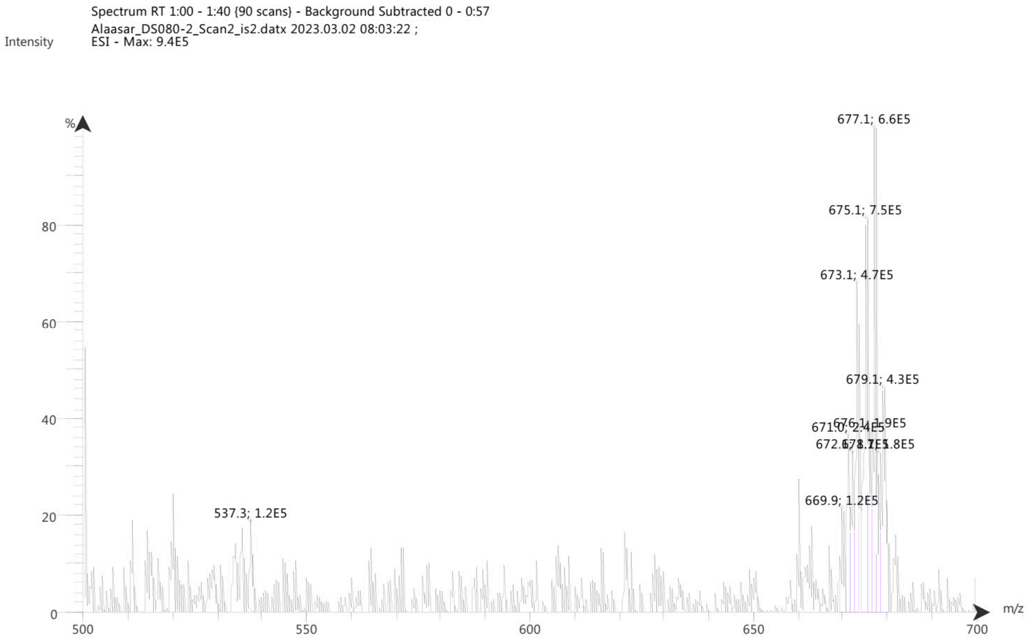

MS chart of compound **4b**

*N,N'-(diselanediylbis(4,1-phenylene))bis(1-(2-nitrophenyl)methanimine) (**4c**)*

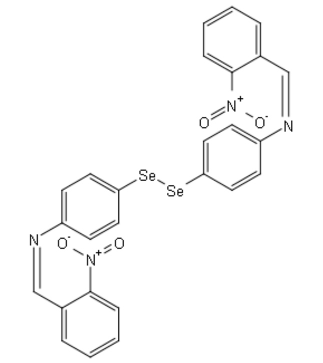

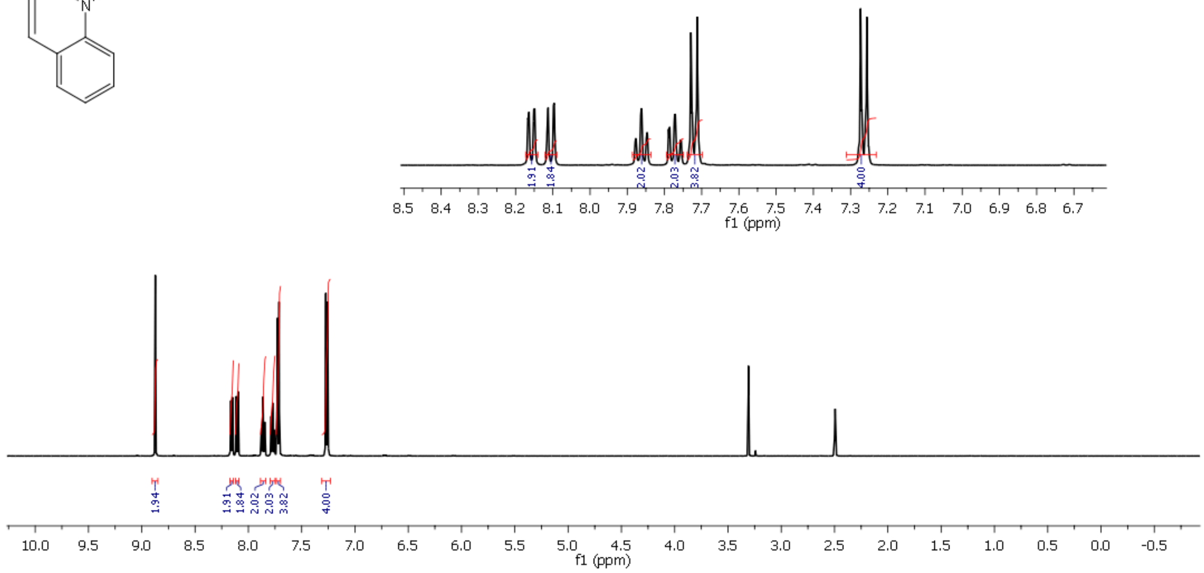

HNMR chart of compound**4c**

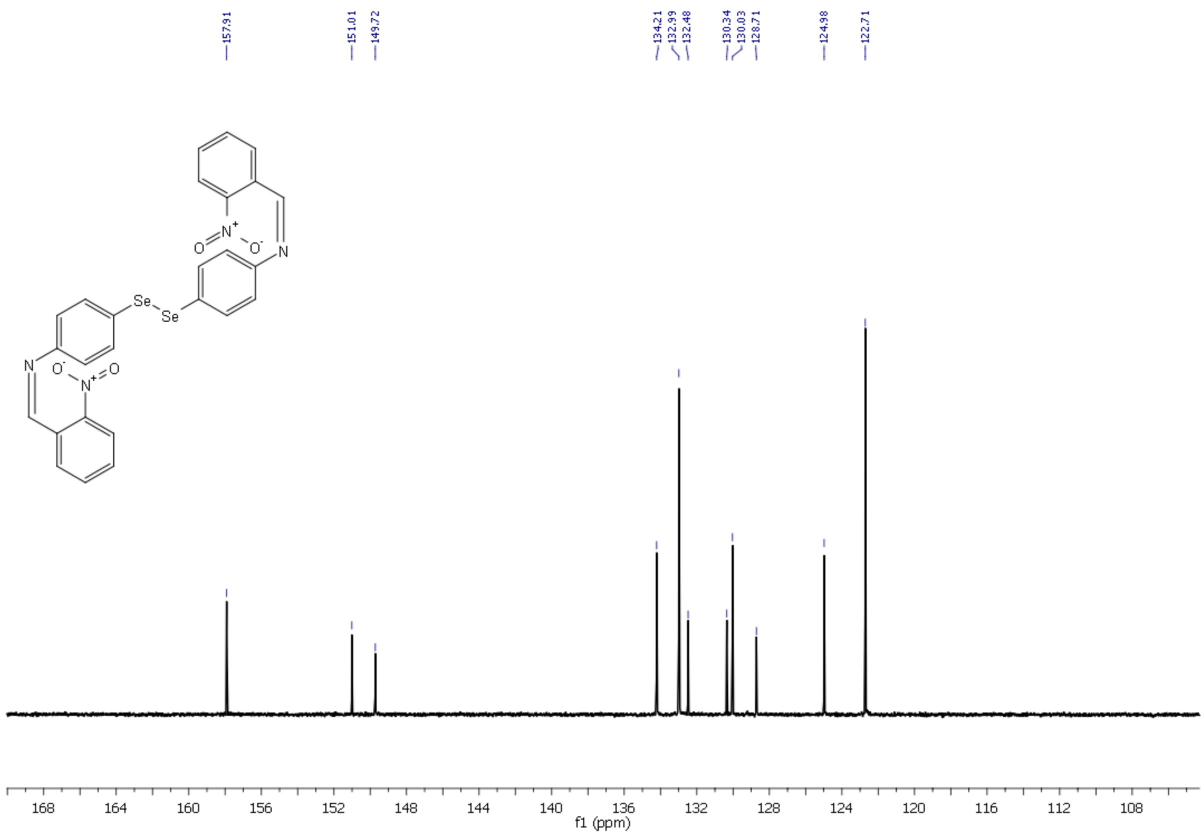

^{13}CNMR chart of compound**4c**

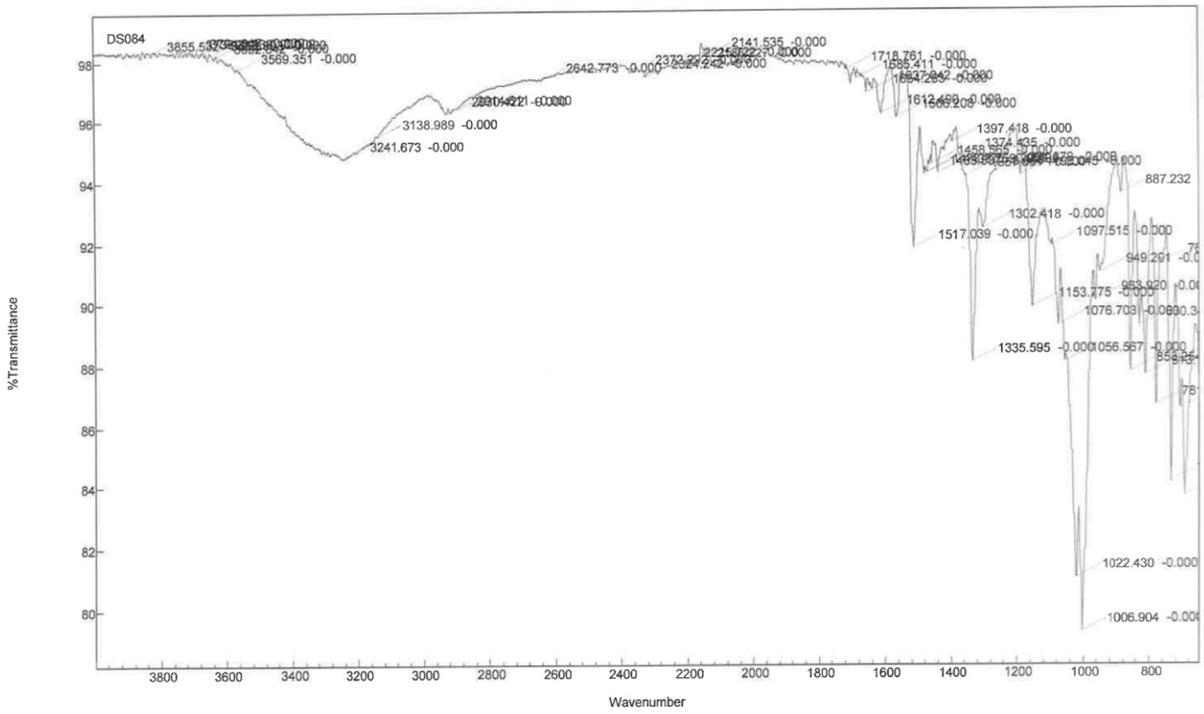

IR chart of compound**4c**

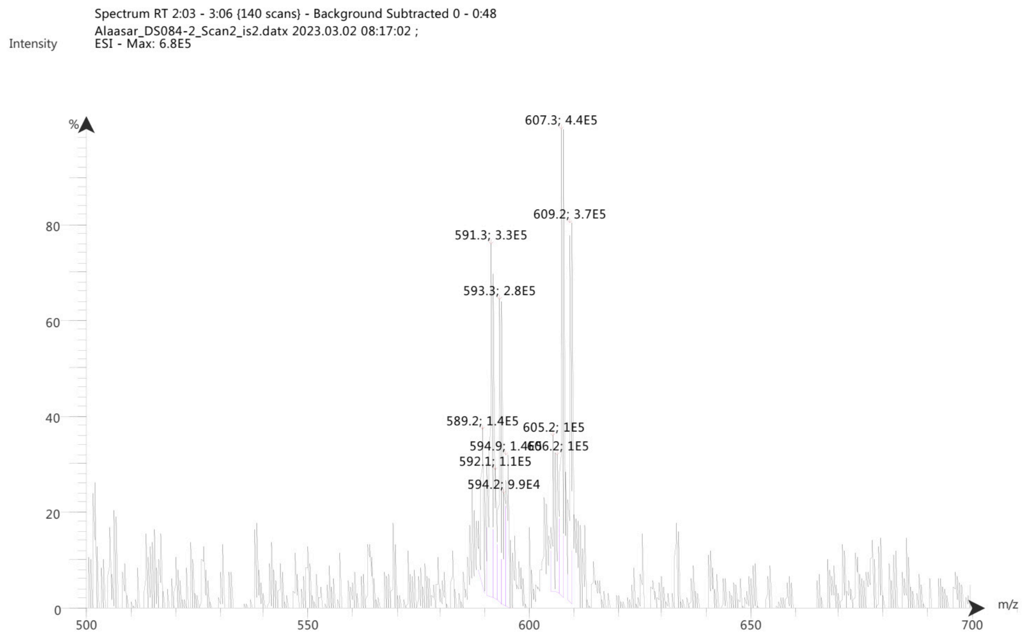

MS chart of compound **4c**

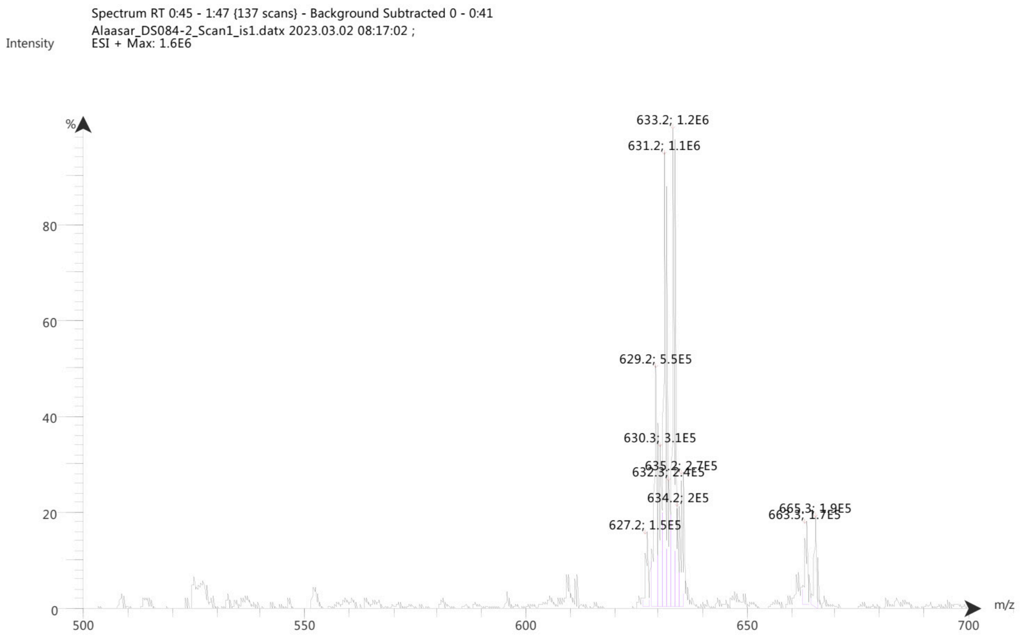

MS chart of compound **4c**

*1-(4-fluorophenyl)-N-(4-(methylselanyl)phenyl)methanimine (**6a**)*

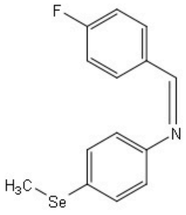

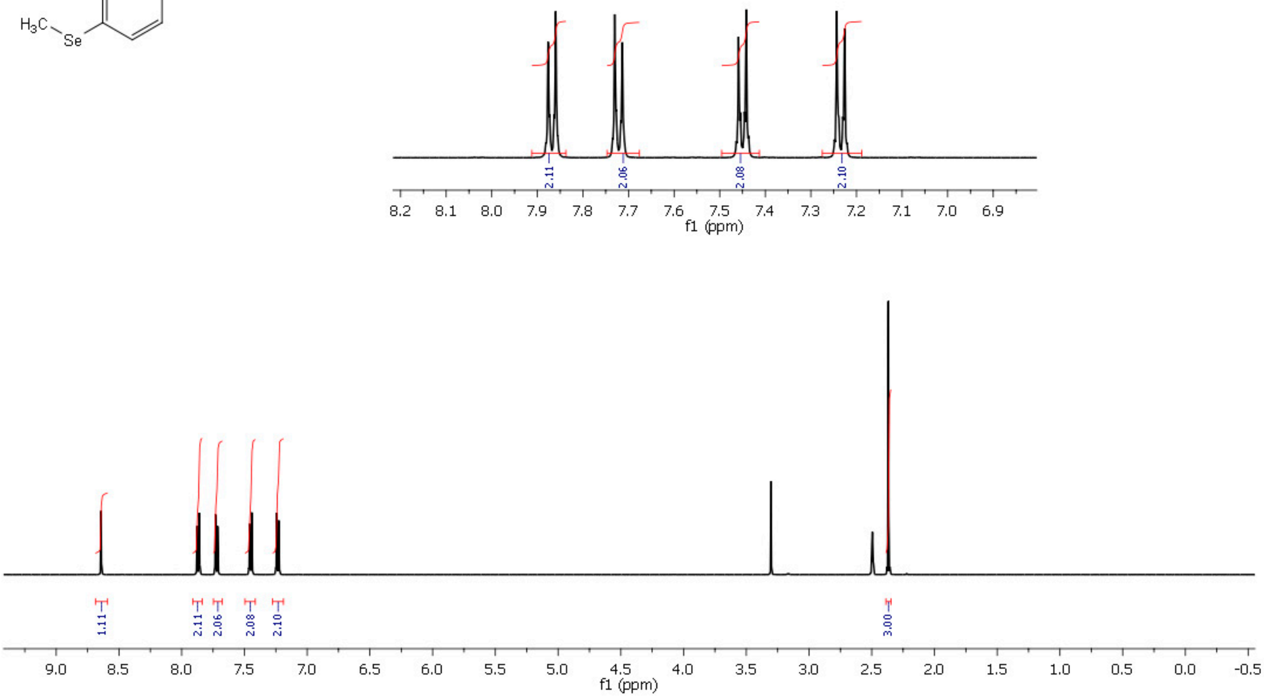

^{1}HNMR chart of compound**6a**

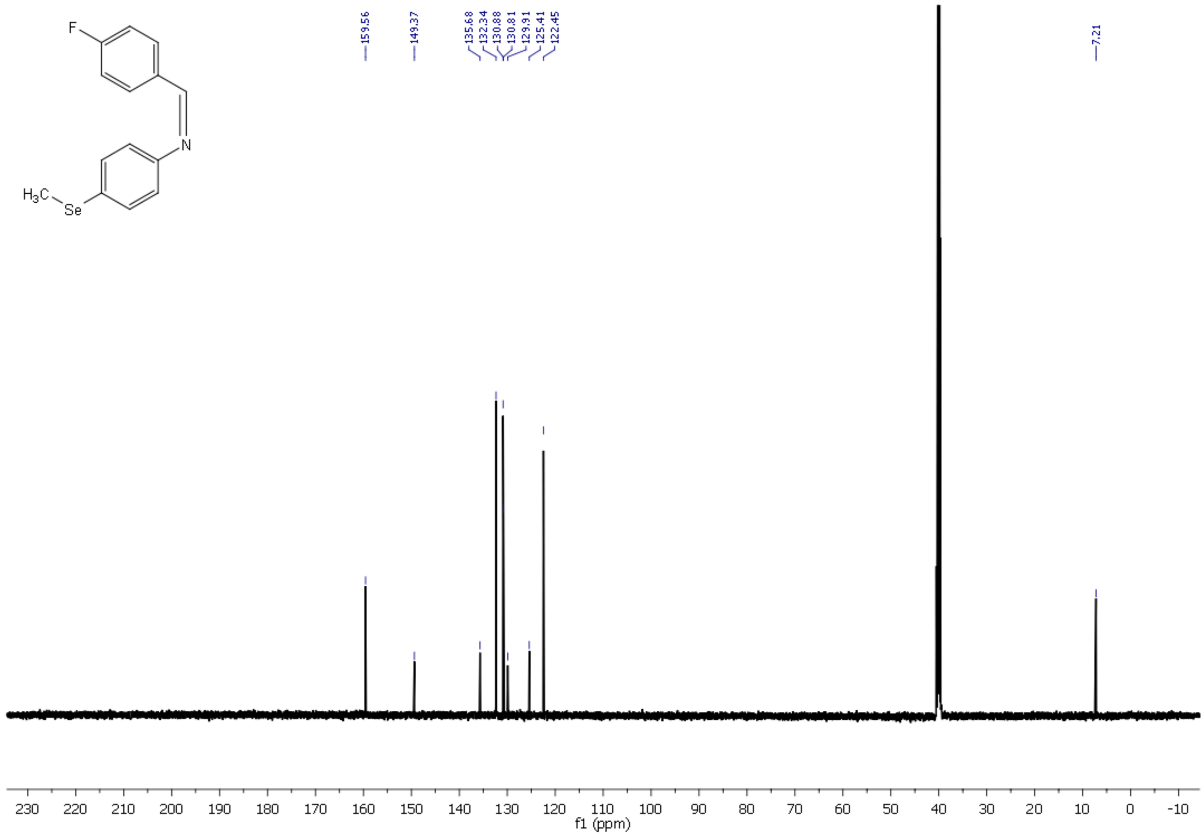

^{13}CNMR chart of compound**6a**

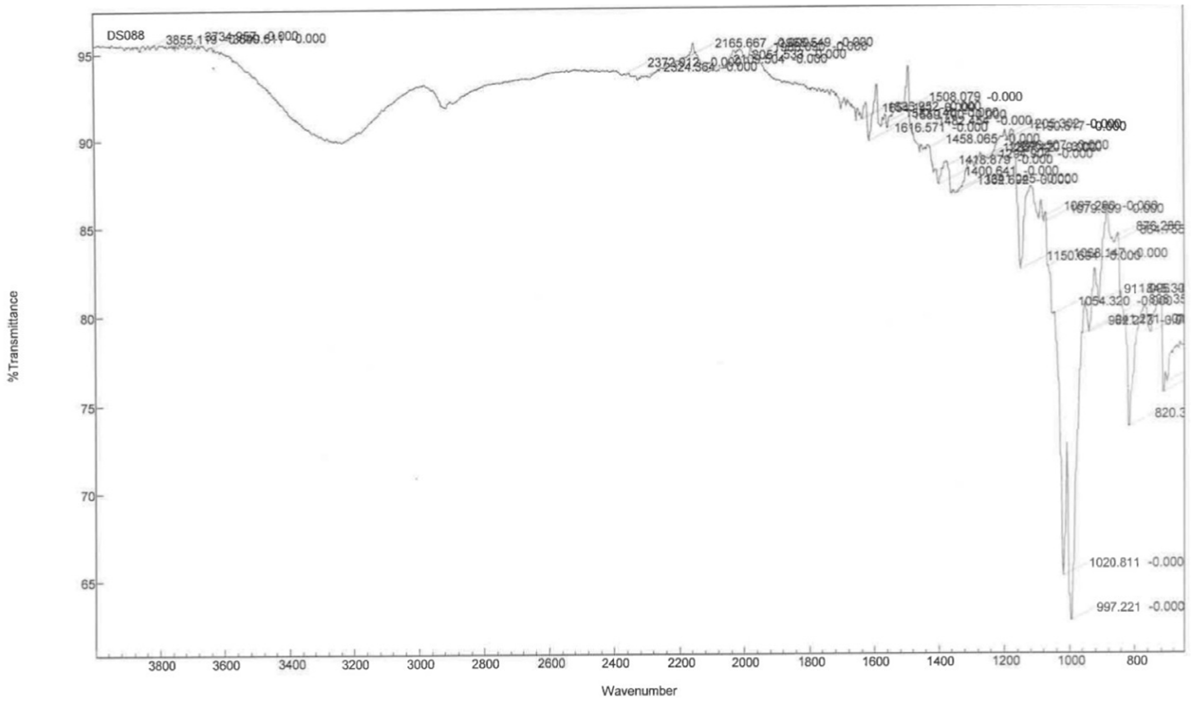

IR chart of compound**6a**

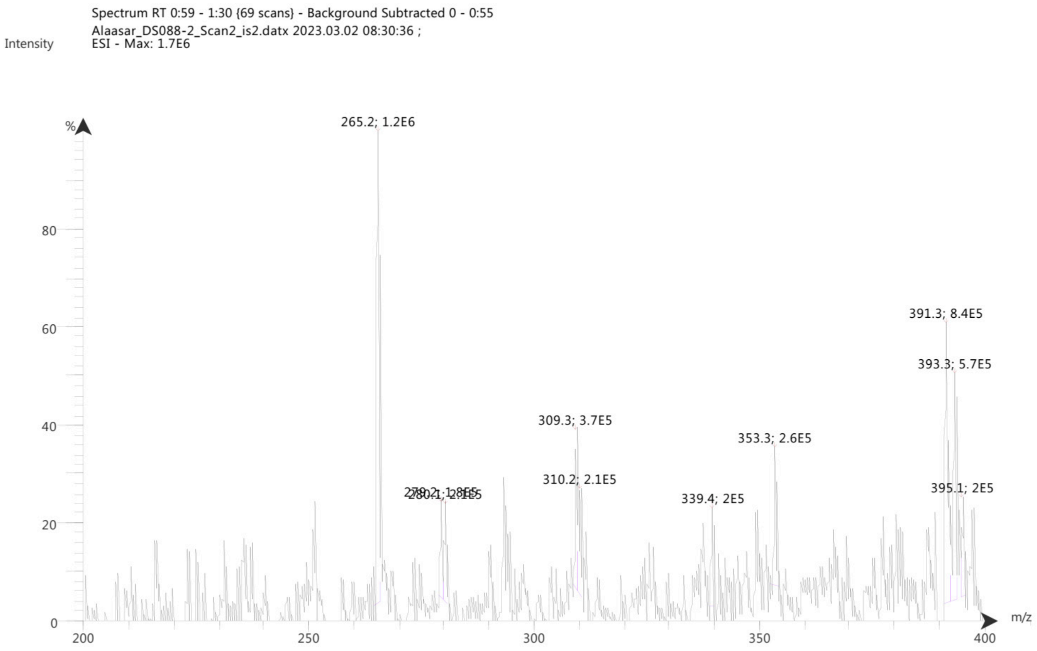

MS chart of compound **6a**

*1-(((4-(methylselanyl)phenyl)imino)methyl)naphthalen-2-ol (**6b**)*

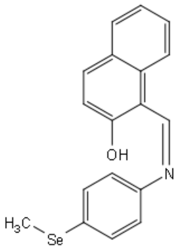

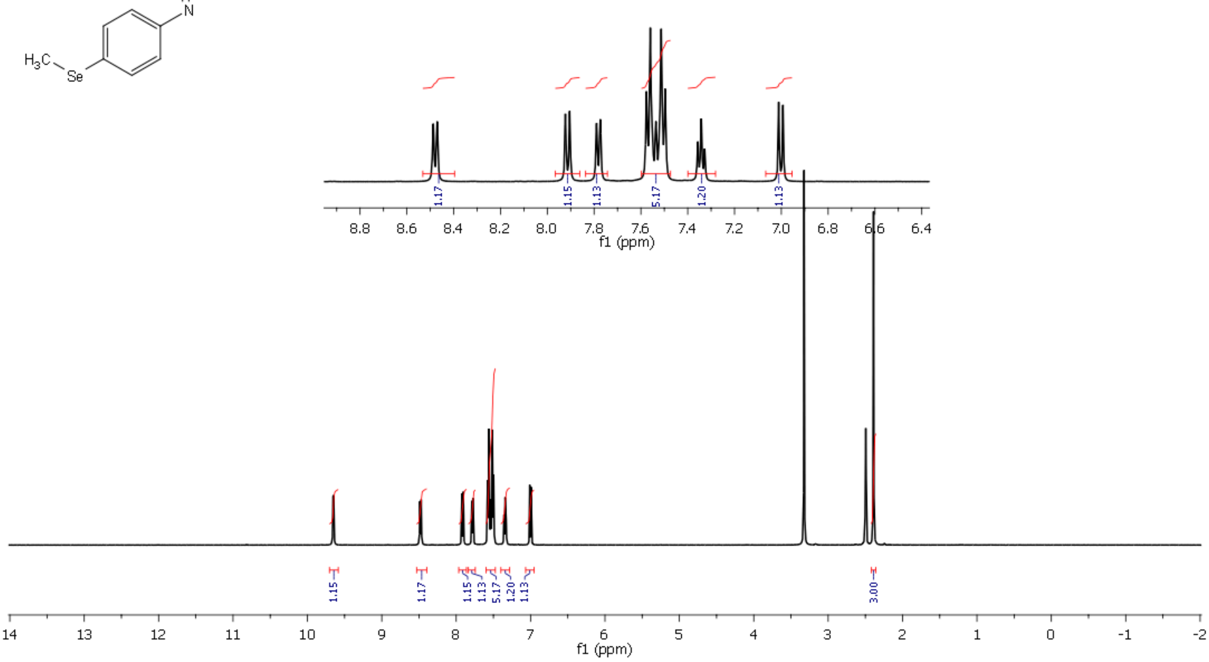

^{1}HNMR chart of compound**6b**

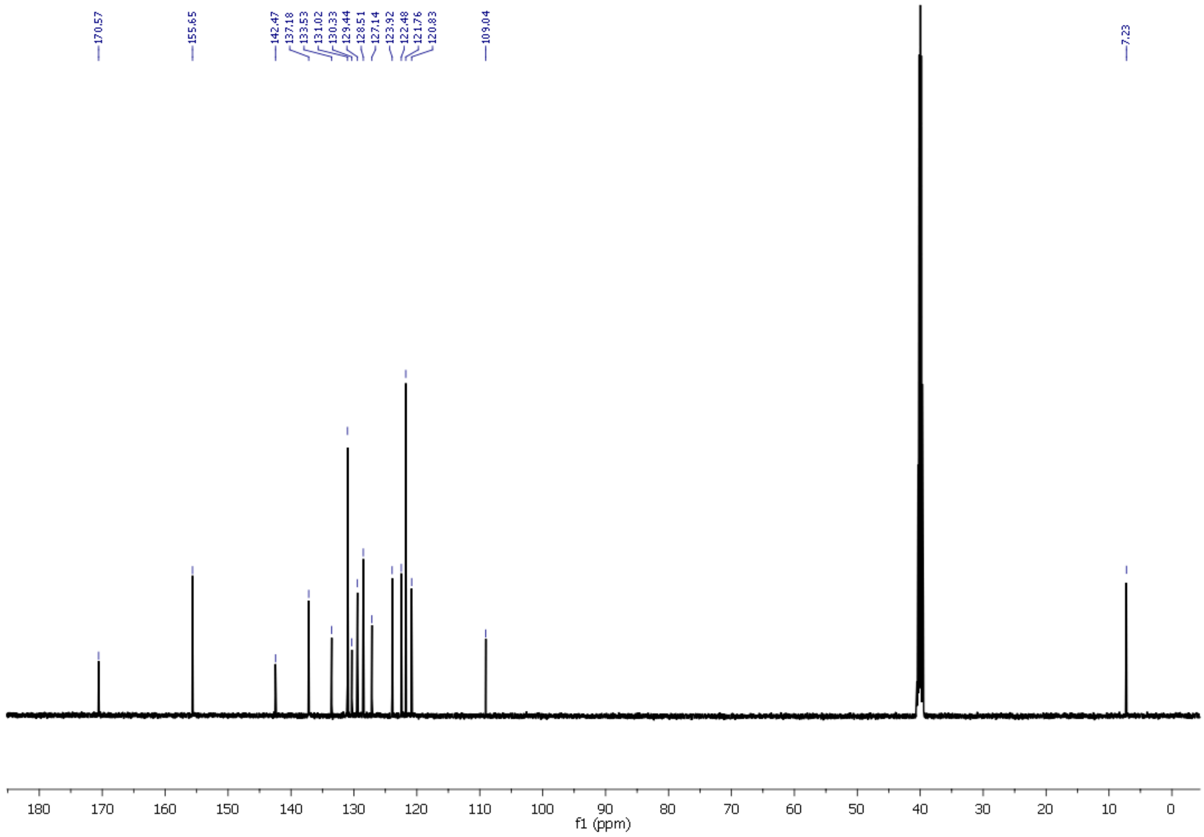

^{13}CNMR chart of compound**6b**

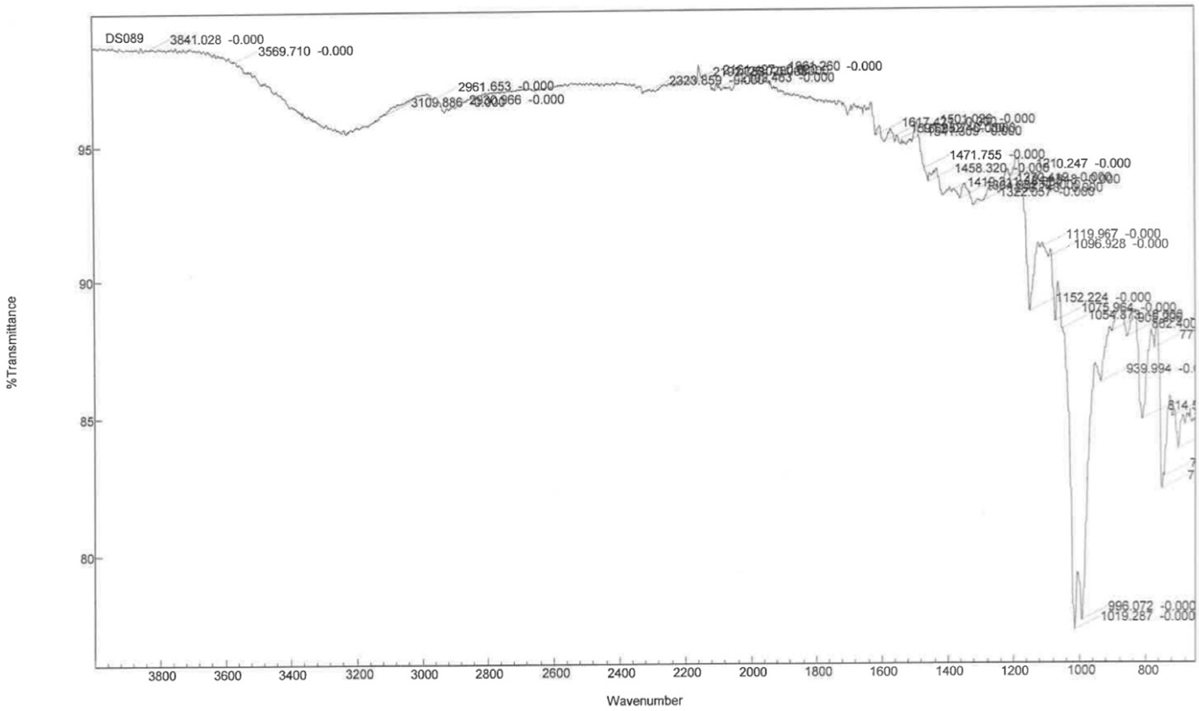

IR chart of compound**6b**

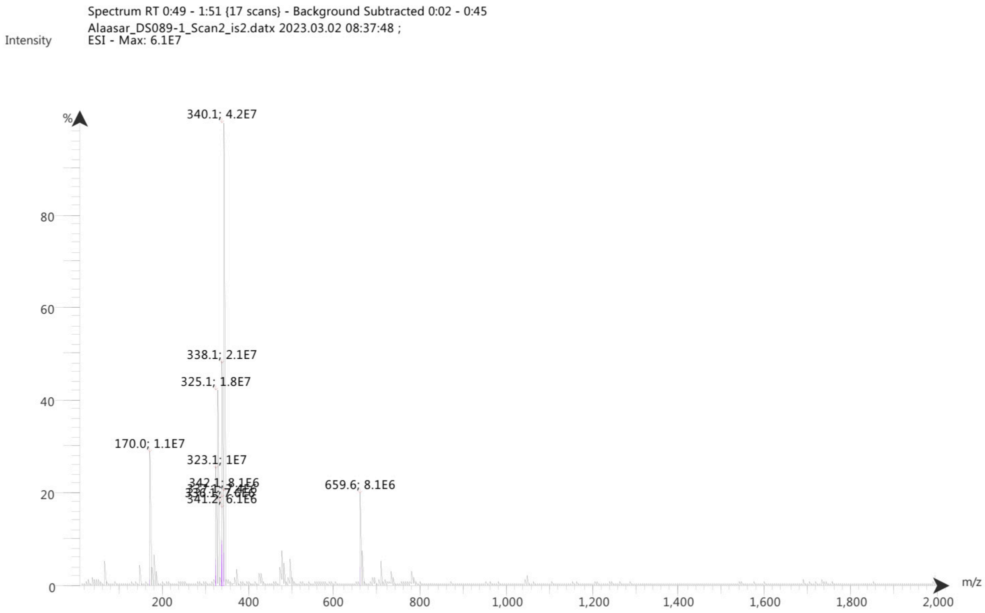

MS chart of compound **6b**

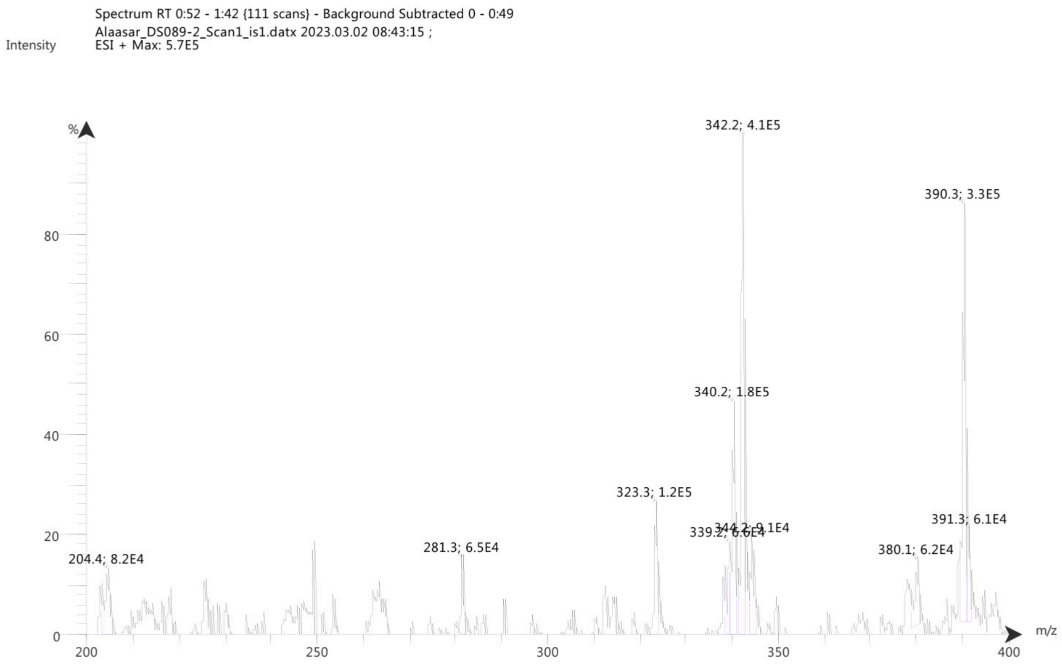

MS chart of compound **6b**

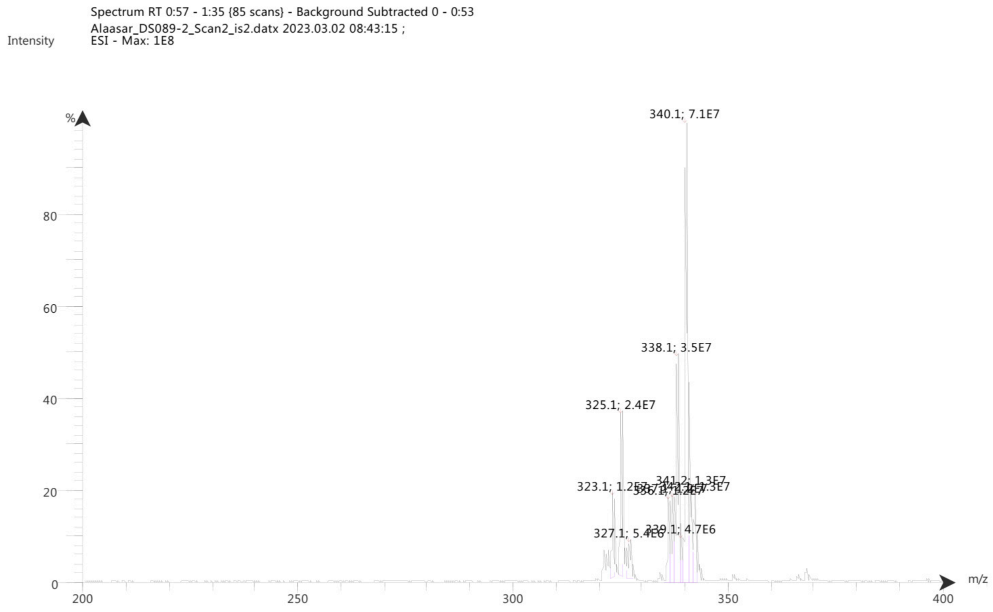

MS chart of compound **6b**

*N-(4-(methylselanyl)phenyl)-1-(2-nitrophenyl)methanimine (**6c**)*

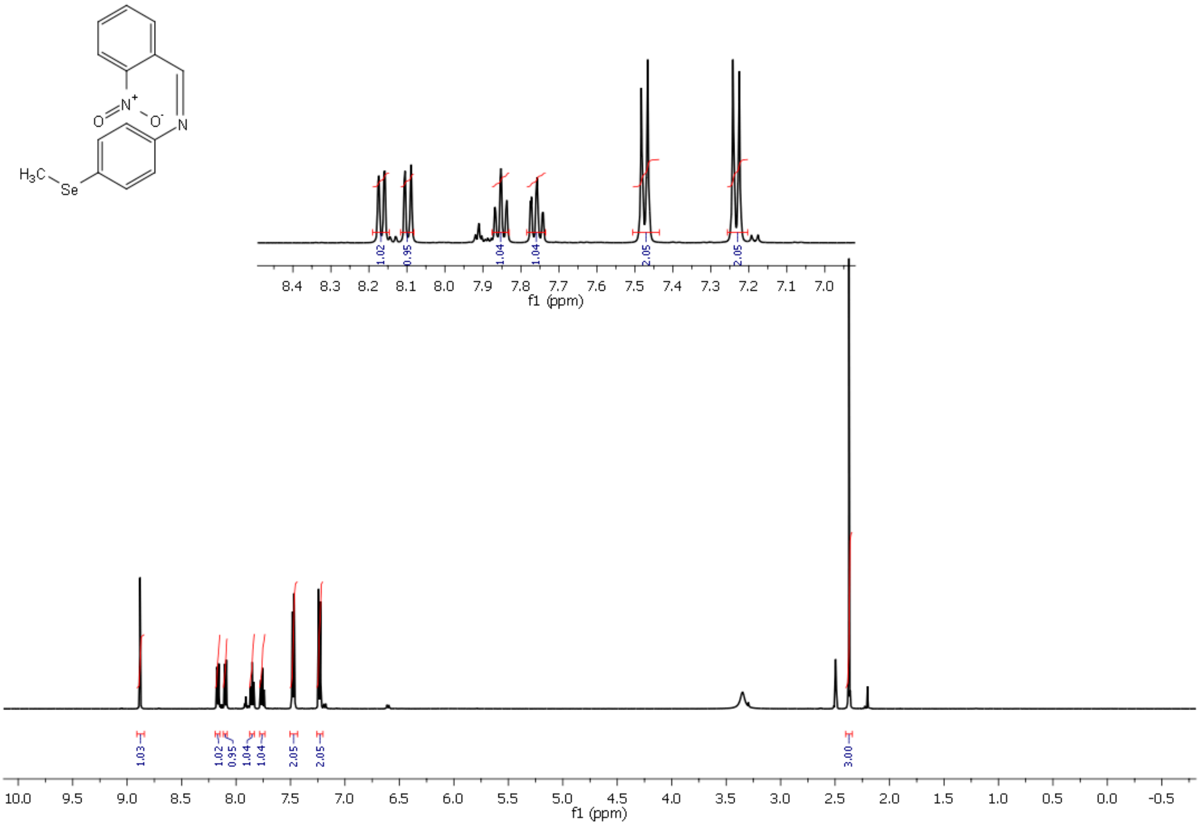

^{1}HNMR chart of compound**6c**

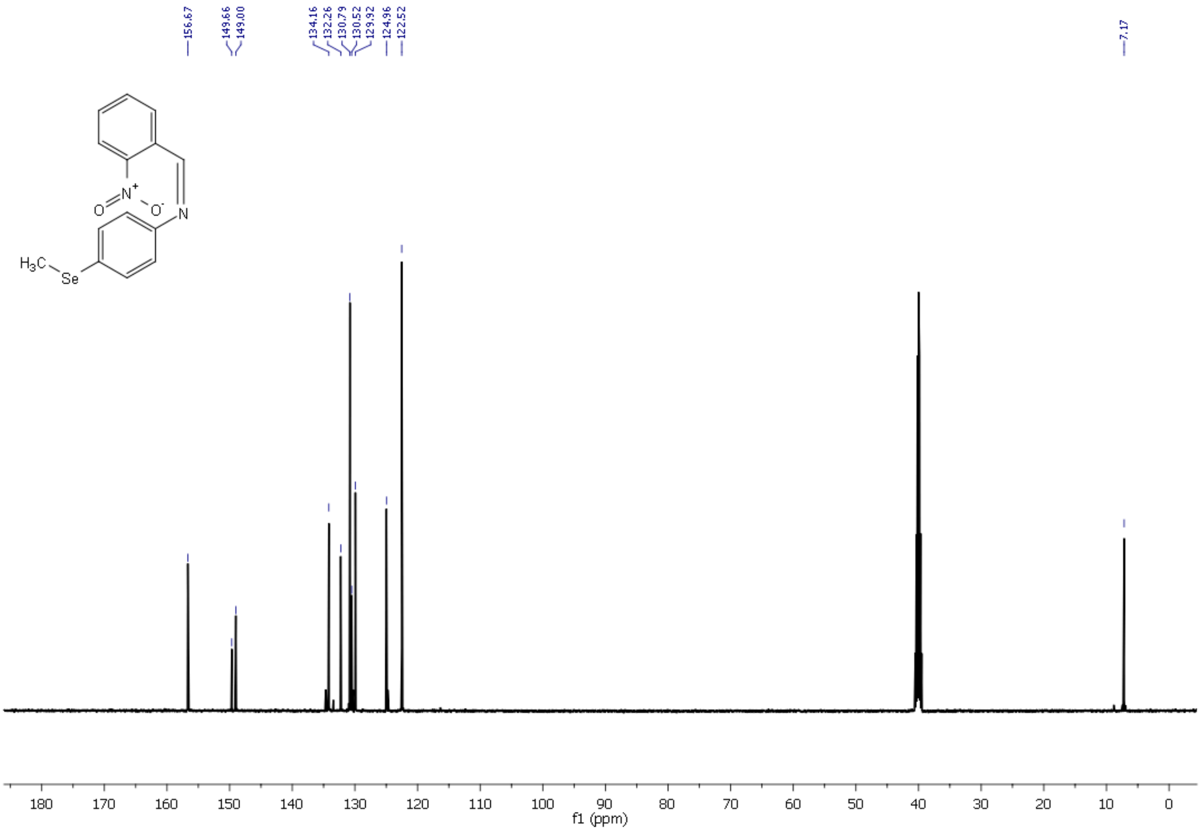

^{13}CNMR chart of compound**6c**

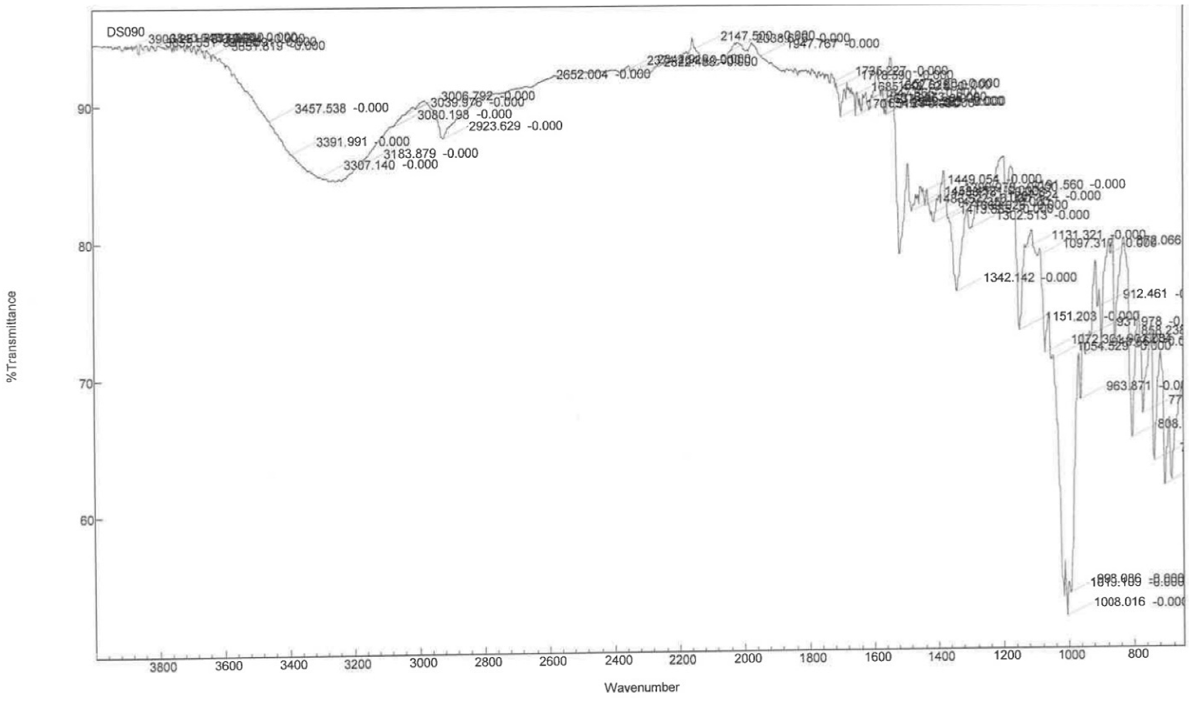

IR chart of compound**6c**

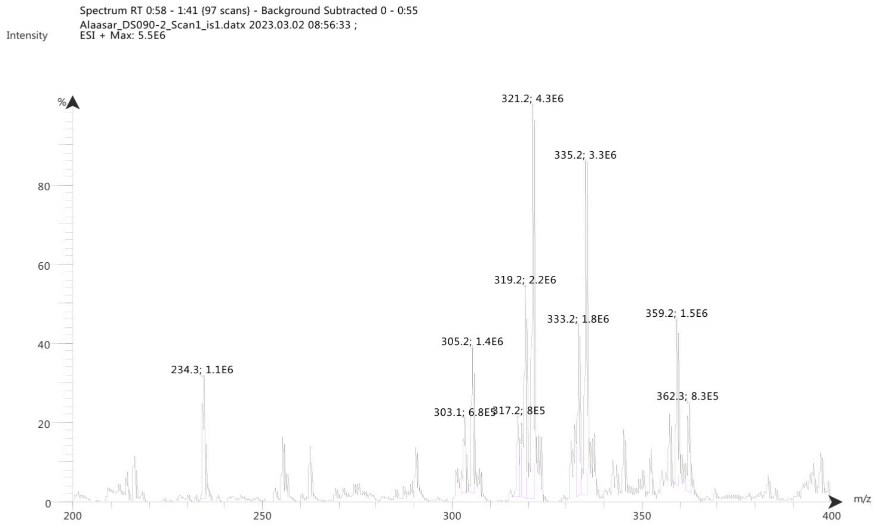

MS chart of compound **6c**
